# An enhanced ensemble defense framework for boosting adversarial robustness of intrusion detection systems

**DOI:** 10.1038/s41598-025-94023-z

**Published:** 2025-04-23

**Authors:** Zeinab Awad, Magdy Zakaria, Rasha Hassan

**Affiliations:** 1 Department of Computer Science, Faculty of Computers and Information Sciences, Mansoura University, Mansoura, 35516 Egypt; 2 Department of Computer Science, Faculty of Computers and Information, Mansoura University, P.O. Box: 35516, Mansoura, Egypt

**Keywords:** Machine learning, Intrusion detection systems, Adversarial attacks, Adversarial defense, Deep learning, Ensemble learning, Mathematics and computing, Computer science, Engineering

## Abstract

Machine learning (ML) and deep neural networks (DNN) have emerged as powerful tools for enhancing intrusion detection systems (IDS) in cybersecurity. However, recent studies have revealed their vulnerability to adversarial attacks, where maliciously perturbed traffic samples can deceive trained DNN-based detectors, leading to incorrect classifications and compromised system integrity. While numerous defense mechanisms have been proposed to mitigate these adversarial threats, many fail to achieve a balance between robustness against adversarial attacks, maintaining high detection accuracy on clean data, and preserving the functional integrity of traffic flow features. To address these limitations, this research investigates and integrates a comprehensive ensemble of adversarial defense strategies, implemented in two key phases. During the training phase, adversarial training, label smoothing, and Gaussian augmentation are employed to enhance the model’s resilience against adversarial perturbations. Additionally, a proactive preprocessing defense strategy is deployed during the testing phase, utilizing a denoising sparse autoencoder to cleanse adversarial input samples before they are fed into the IDS classifier. Comparative evaluations demonstrate that the proposed ensemble defense framework significantly improves the adversarial robustness and classification performance of DNN-based IDS classifiers. Experimental results, validated on the CICIDS2017 and CICIDS2018 datasets, show that the proposed approach achieves aggregated prediction accuracies of 87.34% and 98.78% under majority voting and weighted average schemes, respectively. These findings underscore the effectiveness of the proposed framework in combating adversarial threats while maintaining robust detection capabilities, thereby advancing the state-of-the-art in adversarial defense for intrusion detection systems.

**Supplementary Information:**

The online version contains supplementary material available at 10.1038/s41598-025-94023-z.

## Introduction

The adoption of machine learning (ML) and deep learning (DL) techniques has significantly advanced various domains, including information security, natural language processing, and computer vision. These approaches have enabled the development of applications with exceptional learning capabilities, particularly in classification and regression tasks, achieving demonstrably high accuracy and robust performance. Notable examples include financial systems^1^, healthcare applications^2^, and cybersecurity solutions^3^. In the context of cybersecurity, deep learning-based network intrusion detection systems (NIDS) have emerged as a powerful tool for safeguarding network traffic against security breaches. These systems can identify malicious activities within large and diverse traffic flows with remarkable precision. The unique strengths of deep learning, such as automated feature extraction, non-convex optimization, and end-to-end learning frameworks, enable it to outperform traditional signature-based and conventional machine learning-based approaches in terms of both detection accuracy and scalability^4^. However, despite their potential, deep neural networks (DNNs) are vulnerable to adversarial attacks. These attacks exploit the inherent weaknesses of DNNs, compromising their reliability and effectiveness. Consequently, while deep learning holds great promise for developing advanced intrusion detection systems, the susceptibility of these systems to adversarial threats poses a significant challenge that must be addressed to ensure their robustness and security^5^.

Adversarial attacks aim to deceive machine learning detectors by introducing subtle, carefully crafted perturbations to input samples, known as adversarial examples, during the deployment phase of deep neural networks (DNNs). These adversarial instances exploit vulnerabilities in the model, leading to misclassifications while remaining imperceptible to human observers. The existence of adversarial examples can be attributed to two primary factors^6^. First, there is often a discrepancy between the distribution of the training data and the distribution of real-world data. This mismatch creates a gap between the learned decision boundary of the model and the optimal decision boundary, rendering the model susceptible to adversarial manipulations. Second, the inherent linearity of neural networks, despite their nonlinear activation functions, contributes to their vulnerability. This linearity allows adversaries to generate adversarial examples near the manifold of the training data using gradient-based techniques, such as the fast gradient sign method (FGSM). These factors collectively underscore the challenges in developing robust machine learning models capable of withstanding adversarial threats^16^.

Deep learning-based network intrusion detection systems (NIDS) are threatened by adversarial attacks that compromise their performance. The attacker crafts the adversarial examples by Introducing perturbation into the training or testing input of the DL models causing them to misclassify certain classes (targeted attack) or misclassify the input sample without emphasis on specific class output (untargeted attack). To tackle the situation, several mitigation approaches have been proposed by the defenders to mitigate or defend against such attacks. Among those common approaches are adversarial training^7^, adversarial detection, feature transformation, label and spatial smoothing, adversarial perturbation elimination, and ensemble defense^8^. The purpose of the research is to assess the effect of adversarial evasion attacks introduced at test time on the intrusion detection system classifier performance. Examining the impact of assembling multiple adversarial defense strategies like adversarial training, gaussian augmentation, and label smoothing to promote robustness on deep learning-based intrusion detection decisions at training time. As an additional preprocessing defense, we investigate the effect of input denoising using an unsupervised deep denoising autoencoder to evade the adversarial instances injected into the input samples before being fed into the IDS classifier at the testing time, which is considered a proactive defense to boost the IDS classifier performance.Additinally, using an ensemble defense rather than an individual defense mechanism offers several advantages. These advantages stem from the diversity, robustness, and complementary strengths of multiple defense strategies working together. All the defense mechanisms are evaluated in semi-white box and black box settings. A black box setting is where the attacker does not have any prior knowledge about the trained model architecture or initialization parameters, or even the defense mechanism and only can access the dataset used. In contrast, the attacker has full knowledge of the trained model and the dataset in the white box setting which is an unrealistic assumption. In a semi-white box environment, the attacker has only partial knowledge about the trained model architecture without the training parameters initialization which is considered more realistic. To evaluate the efficacy of the proposed ensemble approach We implement a baseline model as an IDS classifier to measure the impact of the generated adversarial examples on the IDS classifier performance before and after applying the defense approaches.

The proposed work has made the following significant contributions, as outlined below:

- Building a multi-module adversarial defense ensemble technique in the context of intrusion detection systems. The proposed defense ensemble mechanism aggregates multi-source adversarial training, gaussian argumentation retraining, adversarial denoising, and label smoothing that significantly increase IDS classifier robustness against adversarial threats while maintaining the intrusion detector’s accuracy on clean data.
- analyze and provide an assessment of DNN-based IDS effectiveness under adversarial free settings, different state-of-the-art evasion attack scenarios, and adversarial mitigation strategies using the CIC-IDS 2017,CIC-IDS 2018 benchmark datasets.
- Allow an Effective one-time retraining and evaluation of the multi-class IDS detector, instead of considering separate intrusion class retraining or reducing the IDS detector task to binary classification.
- Fulfil the constraint of Preserving the functionality of traffic features during the adversarial generation by Leveraging Extremely Randomized Trees for robust non-functional feature selection.
- This paper provides a thorough theoretical foundation, technical understanding of the suggested ensemble defense strategy with two major stages during the training and testing time by providing step-by-step implementation procedures and algorithms in detail.
- highlight the key benefits of using an ensemble defense mechanism rather than relying on individual defense mechanisms in enhanced robustness against adversarial attacks and improved detection accuracy through the complementary strength of the ensemble members.

The following sections are organized as follows; Section 2. Background provides a brief background about machine learning-based intrusion detection, adversarial attack methods, and adversarial threat model. Whereas most recent literature studies are discussed in Section 3 . Related articles. Section 4. The proposed approach illustrates the proposed two-phase ensemble defense approach. The experimental analysis and results are covered in Section 5. Experimental results and evaluation. The results obtained and their comparison to the existing approaches are discussed in Section 6. Discussion and comparison with related works. Section 7. Conclusion and future work concludes the proposed work and provides recommendations for future work.

## Background

### Intrusion detection systems

An intrusion detection system is a mechanism that attempts to discover or detect abnormal access to computers or intrusions that incorporate unauthorized access, malicious activities, and cyber-attacks^3^. Current IDS face the problems of low detection rates and hence slow response in addition to high false positive and false negative rates due to the dynamic and complex nature of cyber threats. Deep Neural networks (DDN)^4^ have become a powerful approach in cyber security IDS, achieving supreme attack detection and prevention rates with low false positive rates over many classical ML approaches.

### Adversarial threat model

Although machine learning-based intrusion detection system (IDS) models demonstrate effective performance in classifying benign and malicious input instances while reducing false alarms, they remain vulnerable to certain types of attacks, particularly adversarial attacks. Adversarial attacks involve the introduction of subtle, often imperceptible perturbations into input samples, which can deceive the trained models into producing incorrect classifications or invalid responses^15^. These adversarial instances are carefully crafted to exploit the vulnerabilities of machine learning models, undermining their reliability and effectiveness. The optimization objective of adversarial attacks can be formally represented as shown in Eq. (1):

1 $$\:{\varvec{m}\varvec{a}\varvec{x}}_{{{\varvec{\Vert}\varvec{x}}_{\varvec{a}\varvec{d}\varvec{v}}-\varvec{x}\varvec{\Vert}}_{\varvec{p}\le\:\varvec{\varepsilon}}}\varvec{\vert}\varvec{c}\left({\varvec{x}}_{\varvec{a}\varvec{d}\varvec{v}}\right)-\varvec{c}\left(\varvec{x}\right)\mathbf{\vert}$$

Here, ∥⋅∥p denotes the p*-*norm distance metric between the adversarial sample **x**_**adv**_ and the original input **x**. The perturbation must remain within a predefined constraint ϵ, ensuring that the adversarial example **x**_**adv**_ does not deviate significantly from **x**. This constraint is crucial to maintain the adversarial example within the bounds of its original class while maximizing the classification discrepancy between **x**_**adv**​_ and **x**^16^. In the context of network intrusion detection systems (NIDS), adversarial perturbations must also preserve the functionality of network traffic. Therefore, ϵ must be carefully chosen to be sufficiently small to avoid disrupting the traffic flow, yet large enough to induce misclassification by the IDS model. This balance ensures that the adversarial example remains effective without compromising the integrity of the network traffic.

Adversarial attacks in intrusion detection systems (IDS) can be categorized into two primary types based on their timing and objectives. Poisoning attacks occur during the training phase, where adversarial instances corrupt the training data to compromise the model’s learning process. In contrast, evasion attacks occur during the testing or validation phase, where adversarial perturbations are introduced to input samples to deceive the trained model into making incorrect predictions. Despite the growing interest in adversarial attacks and defense mechanisms for intrusion detection models, this area remains in its early stages of research, with significant opportunities for further exploration and development^18^. This paper focuses on evaluating the impact of various gradient-based evasion attacks on the performance of intrusion detection models. Specifically, we examine the **Fast Gradient Sign Method (FGSM)**, **Basic Iterative Method (BIM)**, **Jacobian-based Saliency Map Attack (JSMA)**, **DeepFool (DF)**, and **Projected Gradient Descent (PGD)** as well as the **Carlini & Wagner (C&W)** adversarial attack. Furthermore, we investigate a comprehensive ensemble of defense strategies designed to mitigate the effects of these attacks. The proposed defense ensemble integrates **adversarial training**, **label smoothing**, **Gaussian augmentation**, and **adversarial denoising** to enhance the robustness of the intrusion detection classifier. Adversarial denoising, a preprocessing defense mechanism, relies on transforming input samples to remove adversarial perturbations before they are fed into the model^14^. In this study, we employ a **denoising sparse autoencoder** to achieve this objective, ensuring that the input data is cleansed of adversarial noise while preserving its functional integrity.

### Attack strategies

Adversarial examples generation depends on various adversarial attack algorithms that encompass the fast gradient sign method (FGSM), basic iterative method (BIM), Deep fool (DF), and Jacobian saliency map method (JSMA), and Projected Gradient Descent (PGD) method as well as the Carlini & Wagner (C&W) method.

#### Fast gradient sign method (FGSM)

**FGSM**^9^ calculates the gradients of a loss function (such as categorical cross-entropy or mean-squared error) for the input instance. An adversarial instance is then created by maximizing the loss using the sign of the gradient by multiplying the signed gradient with a small adjustment Ɛ close to the loss function value to ensure the indistinguishable perturbations. The perturbation equation is given by Eq. (2)

2 $$\:\varvec{a}\mathbf{d}{\mathbf{v}}_{\mathbf{x}}=\mathbf{x}+\mathbf{\epsilon}\mathbf{*}\mathbf{s}\mathbf{i}\mathbf{g}\mathbf{n}\left({\nabla\:}_{\mathbf{x}\:}\mathbf{j}\left(\varvec{\uptheta\:},\mathbf{x},\mathbf{y}\right)\right)$$

In Eq. (2), **adv**_**x**_ represents the perturbed variant of the input sample **x**, with predicted class label **y**; **θ** stands for the network weights; and **j(θ**,** x**,** y)** is the loss function that was utilized to train the neural network. To disturb the benign data sample, the sign of the gradient of the loss function is scaled by $\:\mathbf{\epsilon}$ and added.

#### Basic iterative method (BIM)

The **Basic Iterative Method (BIM)** is an iterative extension of the **Fast Gradient Sign Method (FGSM)**, designed to perform a more refined optimization of adversarial perturbations. Unlike FGSM, which applies a single-step perturbation, BIM iteratively applies FGSM with a small step size over multiple iterations. Starting from the original input, BIM incrementally adjusts the perturbation at each step, ensuring that the adversarial example remains within a predefined valid range (bound) by clipping the perturbation after each iteration. This iterative process allows BIM to generate more effective adversarial examples with subtle, carefully controlled modifications, making it a stronger attack compared to the single-step FGSM^10^.

#### The projected gradient descent (PGD)

**PGD** is a more general and powerful iterative attack method. It is used for both targeted and untargeted attacks. It is considered a universal first-order adversary because it is highly effective against many defenses. Like BIM, PGD iteratively applies small perturbations and projects the result back into the valid input space. Apply small perturbations iteratively. Projection Ensures the adversarial example stays within the ϵ-ball around the original input. It is often more effective and stronger than BIM because it starts from a random point with the ϵ-ball, making it more robust to defend.

#### The deepfool (DF)

It is a non-targeted attack introduced by Moosavi Dezfooli et al.^11^. It is one of the attack methods to apply the minimal perturbation for misclassification under the **L**_**2**_ distance metric. The method performs iterative steps on the adversarial direction of the gradient provided by a locally linear approximation of the classifier until the decision hyperplane has crossed.

#### Jacobian-based saliency map attack (JSMA)

Papernote et al.^12^ designed an efficient adversarial attack method called **JSMA** in 2016. The algorithm selects features with the greatest impact on the output target class, called target features, using forward derivative and adversarial saliency maps. It then obtains adversarial examples **x**_**adv**_ by modifying target features. If the adversarial example is unsuccessful in fooling the target DNN, the adversarial saliency map of **x**_**adv**_ is calculated again until the attack is successful.

#### Carlini & Wagner (C&W) adversarial attack

It is a powerful optimization-based method for generating adversarial examples introduced by Nicholas Carlini and David Wagner^30^. It is widely used to evaluate the robustness of machine learning models and to develop defenses against adversarial attacks. By formulating the problem as an optimization task, the **C&W** attack can generate highly effective adversarial examples with minimal perturbation. The C&W attack optimization function could be formulated as follows:

The C&W attack solves the following optimization problem:

3 $$\:\text{min\:}\delta\:|{\updelta\:}{|}_{p}+c\cdot\:f\left(x+{\updelta\:}\right)$$

Subject to: $$\:x+{\updelta\:}\in\:{\left[\text{0,1}\right]}^{n}$$

Where δ is the perturbation added to the input x. ∥δ∥p is The Lp norm of the perturbation (e.g., L_2_, L_∞_) to measure its magnitude, **c** is a hyperparameter that balances the trade-off between minimizing the perturbation and maximizing the effectiveness of the attack. $\:f$(x + δ) is A loss function designed to ensure that the adversarial example x + δ is misclassified by the model.

. The objective is typically a combination of two conflicting objectives:

- Minimizing the perturbation: To ensure that the adversarial example remains visually like the original input.
- Maximizing the misclassification confidence: To guarantee that the perturbed input is misclassified by the target model, and it is controlled by **c** weight.

### Deep autoencoder

Deep autoencoder^14^ is a type of neural network architecture trained in an unsupervised way to learn the manifold of the training samples distribution. The goal is to create an encoded representation of the input by extracting the most relevant aspects from it called latent space representation and then decompress it to reobtain the original input. The autoencoder is composed of two neural networks: an Encoder that compresses the information of the input into a lower dimensional latent code, and a Decoder that decompresses the encoded representation back to the original input.in our work, we use the sparse autoencoder that learns better representations and outperforms the original autoencoder due to L_1_ sparsity regularization which makes its activations sparser.

## Related articles

IDS are implemented to find illicit activity and policy infractions in a system or network. Techniques employed by attackers to deceive or bypass detection systems are known as adversarial attacks. This implies creating perturbed input data for an intrusion detection system (IDS) that deceives the system into not detecting an intrusion. Adversarial attack model threats on IDS encompass evasion, poisonous, and exploratory attacks. To defend against such attacks on the IDS domain researchers and defenders develop several defense mechanisms that are dependent upon data preprocessing, robust machine learning algorithms, feature engineering and selection, anomaly detection, regularization techniques, and adversarial example detection. The defense strategies are applied on different IDS architectures such as RNN, DNN, CNN, AE, LSTM, DRL, and GANs. Most of the research papers in this field reduce the classification task to binary classification rather than multi-classification. The evaluation is performed based on various metrics such as detection accuracy; false positive/negative rates; and robustness metrics using different benchmark datasets^27^.

The two-phase defense strategy against the most potent optimization-based adversarial assault, Carlini & Wagner (C&W), has been suggested by Roshan, MS Khushnaseeb et al.^13^. The training phase and the testing phase are the two defense phases. They employed Gaussian Data Augmentation (GDA) to modify the adversarial training strategy throughout the training phase. The resulting adversarial list was subjected to the Feature Squeezing (FS) approach during the testing phase and then sent to the robust NIDS model for final classification. The suggested two-phase defense strategy is successfully assessed in terms of classification reports and confusion metrics using the most recent CIC-DDoS2019 dataset.

Alper Sarıkaya et al.^14^. Proposed a technique that generates adversarial attack data using generative adversarial networks. Next, they present RAIDS, a strong intrusion detection system (IDS) model that is built to withstand adversarial attacks. The reconstruction error of an autoencoder is utilized in RAIDS as a prediction value for a classifier. Additionally, several feature sets are generated and utilized to train baseline machine learning classifiers, preventing the attacker from speculating on the feature set. The output from two autoencoders and an ensemble of baseline machine learning classifiers is then used to train a LightGBM classifier. The findings demonstrate that, in the absence of adversarial training, the suggested robust model can boost overall accuracy by at least 13.2% and F1-score by more than 110% against adversarial attacks.

Using adversarial training, Matheus P. Novaes et al.^15^ suggested a detection and protection system in an SDN environment. For discovering DDoS attacks the Generative Adversarial Network (GAN) framework was utilized. To ensure that the system is more resilient against adversarial attacks They employ adversarial training. Their system contains well-defined modules to enable traffic monitoring continuously by analyzing the IP traffic flow, allowing the anomaly IDS to act in near-real time. They ran tests on two different situations, using both synthesized data and the publicly available CIC-DDoS 2019 dataset. In comparison to other techniques, experimental results revealed the system’s effectiveness in detecting the newest unknown typical DDoS attacks sorts. They compared the proposed system’s results to those of various neural network architectures for detecting DDoS in SDN that have been published in the literature, like CNN, LSTM, and MLP.

An ensemble defense method specifically created for NIDS; Def-IDS was proposed by Wang et al.^16^ to thwart known as well as undiscovered adversarial attacks. It is a two-module training technique that combines multi-source adversarial retraining with multi-class generative adversarial networks to enhance model robustness while preserving detection accuracy on undisturbed samples. They assess the mechanism using the CSE-CIC-IDS2018 dataset and contrast its output with that of the other three defense strategies. The outcomes show that Def-IDS has superior accuracy, precision, recall, and F1 score when it comes to detecting different hostile attacks.

MACGAN is an anomaly detector attack framework introduced by Zhong et al.^17^. It is composed of two elements. The first element is used to manually assess the attack feature fields. Then, in the second element, the GAN’s used to deceive the anomaly detection process. To evaluate their approach, The Kitsune2018 and CIC-IDS2017 data sets were utilized. The capability to evade the latest common machine learning classifiers has been demonstrated in experiments. This considerably aids security researchers in improving the detector’s stability.

R. Abou Khamis et al.^18^ employ a min-max strategy for analyzing and evaluating the robustness of various forms of DNN-based IDS against a variety of adversarial threats including FGSM, CW, BIM, PGD, and DEEPFOOL. They constructed 36 structures to demonstrate that the adversarial training-based min-max strategy is considered a reliable protection in deep learning-based IDS.

AMBER (Autoencoder and Model-Based Elimination of features using Relevance and Redundancy scores) was introduced by Sharan Ramjee and Aly El Gamal^19^. It used two models, namely the ranker model and the autoencoder. The current model aims to eject correlated features, while the earlier model concentrates on ejecting features that are not essential for classification tasks. It compromises between filter and wrapper methods, which have efficient computation and high performance, respectively.

In their study, Prasad and Chandra^28^proposed the VMFCVD framework, an optimized machine learning-based approach to ensure effectiveness in detecting and mitigating volumetric DDoS attacks. The authors emphasized the importance of preprocessing network traffic data to improve the performance of their machine-learning models. Key preprocessing steps included are (1)Data Cleaning which involves handling missing values, removing duplicates, and filtering irrelevant traffic.(2)Feature Extraction which involves Deriving statistical, flow-based, and time-based features from raw network traffic data. (3)Feature Selection based on correlation analysis and machine learning techniques (e.g., Recursive Feature Elimination) to identify the most relevant features for DDoS detection.(4)Data Normalization by scaling features using min-max normalization or z-score standardization(5)Handling Imbalanced Data by Addressing class imbalance through oversampling techniques like SMOTE. (6)Data Splitting by dividing the dataset into training, validation, and test sets while maintaining class distribution. (7)Data Encoding by encoding categorical features into numerical formats using one-hot or label encoding. These preprocessing steps ensured that the data was clean, normalized, and suitable for training machine learning models, ultimately leading to high detection accuracy and low false positive rates for volumetric DDoS attacks.

In their work, Prasad and Chandra^29^ introduced Bot Defender, a collaborative framework for detecting and mitigating botnet attacks using machine learning and network traffic analysis. The authors highlighted the critical role of the preprocessing stage in preparing network traffic data for effective machine learning model training. The main preprocessing steps included are Data Cleaning: which involves Handling missing values, removing duplicates, and filtering out irrelevant or benign traffic. Feature Extraction: accomplished by Deriving statistical, flow-based, and behavioral features (e.g., packet counts, flow duration, connection frequency) from raw network traffic data. Feature Selection: Using correlation analysis and machine learning-based techniques (e.g., Recursive Feature Elimination) to identify the most relevant features for botnet detection. Data Normalization: Scaling features using techniques like min-max normalization or z-score standardization. Handling Imbalanced Data: Addressing class imbalance through oversampling techniques such as SMOTE or under-sampling methods. Data Splitting: Dividing the dataset into training, validation, and test sets while maintaining class distribution. Data Encoding: Converting categorical features (e.g., protocol type) into numerical formats using one-hot encoding or label encoding. Data Transformation: Reshaping data into a format suitable for machine learning models, including creating time-series windows for sequential analysis. These preprocessing steps ensured that the data was clean, normalized, and ready for training machine learning models, resulting in high detection accuracy and robustness against botnet attacks.

## The proposed approach

This Section explains the proposed ensemble defense approach to mitigate and defend against multi-source adversarial examples. It presents the theoretical foundation of the ensemble defense model components encompassing formal derivation for the optimization function of each defense method and the overall optimization function of the ensemble defense model. The suggested defense approach is implemented in two main stages. First, at the training time, by retraining the DNN-based IDS classifier with adversarial examples in adversarial training, data samples were augmented with Gaussian noise in Gaussian augmentation and data samples with the smoothed label in the label smoothing technique. The purpose of this stage is to boost the robustness of the target IDS classifier against adversarial threats. Second, at the inference time denoising or purifying the input samples to face and mitigate the effect of adversarial examples using sparse denoising autoencoder. We begin by building and training a deep learning-based intrusion detection system on the CIC-IDS2017 dataset^20^ clean samples. Next, both before and after building the adversarial ensemble defense, the deep learning-based IDS’s robustness to adversarial examples is investigated and confirmed. We generate multi-source adversarial samples based on the substitution-trained classifier and evaluate their impact on the DNN-based IDS classifier without the use of any adversarial defense. The following techniques were used to create adversarial samples for this study: the Fast Gradient Sign Method (FGSM), DeepFool (DF), Basic Iteration Method (BIM), Jacobian-based Saliency Map Attack (JSMA), projected gradient descent (PGD), Carlini and Wagner (C&W).

This research investigates the following key propositions: Adversarial attacks are executed during the inference phase of the deep neural network (DNN)-based intrusion detection system (IDS) model, specifically focusing on evasion attacks. We assume that the attacker has limited or no knowledge of the IDS model’s internal architecture and parameters, thereby considering both semi-white box and black box attack scenarios. Furthermore, the adversarial attacks under examination are untargeted, meaning the objective is not to misclassify input samples into a specific target class but rather to deceive the DNN-based IDS classifier into producing incorrect predictions. Because of these attacks, we anticipate a degradation in the performance of the DNN-based IDS classifier, as evidenced by a decline in various performance metrics.

**Fig. 1:**
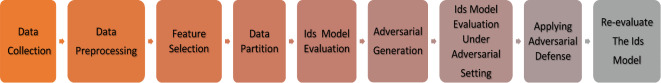
The Study Framework.

Figure 1 illustrates the framework of this study, which consists of nine sequential stages. The process begins with the collection of the CIC-IDS dataset, followed by data preprocessing to clean and prepare the data for analysis. Next, the Extremely Randomized Trees (ERTs) feature selection technique is applied to identify and retain the most relevant and functional traffic features while excluding redundant or irrelevant ones. The dataset is then split into training and testing sets to facilitate model development and evaluation. Subsequently, the intrusion detection system (IDS) model is trained, and its performance is evaluated on clean data. Adversarial examples are then generated to simulate potential attack scenarios, and the IDS model is re-evaluated under these adversarial conditions to assess its vulnerability. To mitigate the impact of adversarial attacks, the proposed adversarial ensemble defense is applied, incorporating multiple defense strategies to enhance model robustness. Finally, the IDS model is re-evaluated to measure the effectiveness of the defense mechanisms in improving its performance under adversarial settings.

To evaluate the effectiveness of each defense technique—adversarial training, Gaussian augmentation, label smoothing, and denoising autoencoder—and to assess their combined impact within the proposed framework, we conduct an ablation study. This study involves training and testing the intrusion detection system (IDS) model with each defense technique applied individually, followed by a comprehensive evaluation of their performance. The results are then compared against the performance of the ensemble approach, which integrates all defense mechanisms. The primary objective of this ablation study is twofold: (1) to determine the standalone effectiveness of each defense technique in mitigating adversarial threats, and (2) to demonstrate the synergistic advantages of combining these techniques into a unified defense mechanism, thereby enhancing the overall robustness and resilience of the IDS model.

### Problem formulation

Let:$\:\mathcal{D}=\{\left({x}_{i},{y}_{i}\right){\}}_{i=1}^{N}\:$​ be the training dataset, where xi is the input and yi is the corresponding label.fθ​ be the model with parameters θ.L(f_θ_(x), y) be the loss function (e.g., cross-entropy loss).The goal is to train a robust model fθ​ that minimizes the classification loss of both clean and adversarial inputs.so the threat model is formalize as:

$$\:{x}_{\text{adv}}=x+{\updelta\:},\hspace{1em}{\updelta\:}=\text{arg}\underset{\left|{\updelta\:}\right|\le\:{\epsilon}}{\text{max}}\mathcal{L}\left({f}_{{\uptheta\:}}\left(x+{\updelta\:}\right),y\right)$$

where $\:\mathcal{D}=\{\left({x}_{i},{y}_{i}\right){\}}_{i=1}^{N}\:$​ be the training dataset, where x_i_ is the input and y_i_ is the corresponding label. F_θ_​ be the model with parameters θ. L(f_θ_(x), y) be the loss function.

### Theoretical foundation for ensemble defense mechanisms

Adversarial training, Gaussian augmentation, label smoothing, and denoising autoencoders (DAEs) are complementary defense mechanisms that enhance the robustness of machine learning models against adversarial attacks. Each technique addresses different aspects of the adversarial vulnerability problem, and their combination creates a more robust and generalized defense. Below is a theoretical foundation explaining their synergy and contribution to adversarial robustness of the model, along with formal derivation and mathematical modeling of the ensemble defense’s optimization function.

**Adversarial training** involves augmenting the training data with adversarial examples x adv, which are generated by perturbing the input data to maximize the model’s loss. This forces the model to learn robust features that are less sensitive to small perturbations.so as a contribution to robustness The model learns to be robust against worst-case perturbations. The adversarial training objective function can be written as:

$$\:\underset{{\uptheta\:}{E}_{\left(x,y\right)\sim\:\mathbb{D}}}{\text{min}}\left[\underset{\left|{\updelta\:}\right|\le\:{\epsilon}}{\text{max}}\mathcal{L}\left({f}_{{\uptheta\:}}\left(x+{\updelta\:}\right),y\right)\right]$$

where:

**f**_**θ**_ is the model with parameters **θ**,**δ** is the adversarial perturbation,ϵ is the perturbation bound, **L** is the loss function (e.g., cross-entropy loss).The min-max optimization ensures the model is robust to worst-case perturbations.

**Gaussian augmentation** adds random Gaussian noise $\:\:\eta\:\:\:$to the input data during training. This helps the model generalize better and become more robust to small input variations.

For a given input x, the augmented input x ~ is:$\:\stackrel{\sim}{x}=x+\eta\:,\hspace{1em}\eta\:\sim\:\mathcal{N}\left(0,{\sigma\:}^{2}I\right)$

where σ controls the noise level,$\:\eta\:\:is\:the$ Gaussian noise with variance σ_2_.the training objective becomes:

$$\:\underset{\theta\:}{\text{min}}{E}_{\left(x,y\right)\sim\:D,\eta\:\sim\:\mathbb{N}\left(0,{\sigma\:}^{2}I\right)}\left[L\left({f}_{\theta\:}\left(x+\eta\:\right),y\right)\right]$$

The overall goal of this is to find the optimal model parameters θ that minimize the average loss over the data samples (x, y) that are drawn from distribution D, considering the perturbations introduced by Gaussian noise $\:\eta\:$. This encourages the model to learn features that are invariant to small noise.so as contribution to robustness it Improves generalization and robustness to small input variations.

**Label smoothing** replaces hard labels (e.g., one-hot encoded vectors) with smoothed labels, which distribute some probability mass to other classes. This reduces overconfidence and improves generalization.

For a label y (one-hot encoded), the smoothed label y~​ is given by:

$$\:\stackrel{\sim}{y}=\left(1-\alpha\:\right)y+\frac{\alpha\:}{K}$$

where α is the smoothing parameter and K is the number of classes. The loss function becomes:

L(f θ(x), y~) =$$\:{\underset{\theta\:}{\text{m}\text{i}\text{n}}\:E}_{\left(x,y\right)\sim\:\mathbb{D}}\left[\mathcal{L}\left({f}_{{{\uptheta\:}}_{2}}\left(x\right),\stackrel{\sim}{y}\right)\right]$$

a general form of the optimization function that incorporates label smoothing:

$$\:L\left(\theta\:\right)=-\frac{1}{N}{\sum\:}_{i=1}^{N}{\sum\:}_{j=1}^{K}\stackrel{\sim}{{y}_{ij}}\text{log}\left({p}_{ij}\right)$$

Where: L(θ) is the loss function with label smoothing represents the parameters of the IDS model. N is the number of samples. K is the number of classes.$\:\stackrel{\sim}{{y}_{ij}}$ is the smoothed label for the i-th sample and the j-th class. $\:{p}_{ij}$is the predicted probability for the i-th sample and the j-th class.

By optimizing this loss function with label smoothing, the IDS model can become more robust to adversarial perturbations, as it reduces the model’s confidence on any single class and encourages it to be more aware of the other classes. This reduces the model’s sensitivity to adversarial examples by preventing overfitting hard labels.so as a contribution to robustness it Reduces overconfidence and improves generalization, making the model less sensitive to adversarial examples, and hence encourages the model to learn more generalized decision boundaries.

**A denoising autoencoder (DAE)** is trained to reconstruct clean inputs from perturbed inputs (with adversarial examples) at the testing time, the optimization function will focus on minimizing the reconstruction error between the clean input x and the reconstructed **output g**_**ϕ**_**(x**_**adv**_**)**, where x_adv_​ is the adversarial example.

Let g_ϕ_ be the DAE with parameters ϕ. The DAE is trained to minimize the reconstruction error between the clean input x and the reconstructed output g_ϕ_(x _adv_) according to the following optimization function:

$$\:\underset{{\upvarphi\:}{E}_{\left(x,y\right)\sim\:\mathbb{D}}}{\text{min}}\left[|{g}_{{\upvarphi\:}}\left(x{+x}_{\text{adv\:}}\right)-x{|}^{2}\right]$$

Where x is the clean input, x_adv_ = x + δ is the adversarial example, with δ being the adversarial perturbation bounded by ϵ, G_ϕ_ is the DAE with parameters ϕ.∥g_ϕ_(x_adv_) − x∥^2^ is the reconstruction error (e.g., mean squared error).The reconstruction error is measured using the squared L2 norm (mean squared error).The optimization function takes the expectation over the dataset D, ensuring that the DAE generalizes well to all inputs.so as contribution to robustness it Removes adversarial perturbations from the input, and hence improving robustness at testing time.

At testing time, the input x is preprocessed by the DAE:

$$\:x\sim={g}_{{\upvarphi\:}}\left(x\right)$$

which reduces the effect of adversarial perturbations before the input is passed to the model.

### Ensemble defense optimization function

The ensemble defense model combines the strengths of all four mechanisms into a unified framework, ensuring the model is resilient to adversarial attacks in IDS.

The overall model optimization function can be modeled as:

**For the training phase**:

$$\:\underset{{{\uptheta\:}}_{1},{{\uptheta\:}}_{2},{{\uptheta\:}}_{3}}{\text{min}}\left[{{\lambda\:}_{1}E}_{\left(x,y\right)\sim\:\mathbb{D}}\left[\underset{\left|{\updelta\:}\right|\le\:{\epsilon}}{\text{max}}\mathcal{L}\left({f}_{{{\uptheta\:}}_{1}}\left(x+{\updelta\:}\right),\stackrel{\sim}{y}\right)\right]+{\lambda\:}_{2}{E}_{\left(x,y\right)\sim\:\mathbb{D}}\left[\mathcal{L}\left({f}_{{{\uptheta\:}}_{2}}\left(x\right),\stackrel{\sim}{y}\right)\right]+{{\lambda\:}_{3}E}_{\left(x,y\right)\sim\:\mathbb{D},{\upeta\:}\sim\:\mathbb{N}\left(0,{{\upsigma\:}}^{2}I\right)}\left[\mathcal{L}\left({f}_{{{\uptheta\:}}_{3}}\left(x+{\upeta\:}\right),\stackrel{\sim}{y}\right)\right]\right]$$

The weighting hyperparameters (λ_1_,λ_2_,λ_3_) are used to balance the contributions of each term. The use of y_smooth (_$\:\stackrel{\sim}{y})$ ensures the model learns smoother decision boundaries and reduces overconfidence during the entire training phase.

**For the testing phase**:

At testing time, the input is preprocessed by the DAE $\:{\varvec{g}}_{\varvec{\varphi\:}}$ and given by the following:

$$\:\stackrel{\sim}{\varvec{x}}={\varvec{g}}_{\varvec{\varphi\:}}\left(\varvec{x}\right)$$

The model obtains the prediction from each model as:

$$\:\widehat{{\varvec{y}}^{1}}={\varvec{f}}_{{\varvec{\uptheta\:}}_{1}}\left({\varvec{x}}_{\varvec{a}\varvec{d}\varvec{v})}\right)\:,\hspace{1em}\widehat{{\varvec{y}}^{2}}={\varvec{f}}_{{\varvec{\uptheta\:}}_{2}}\left({\varvec{x}}_{\varvec{a}\varvec{d}\varvec{v})}\right)\:,\hspace{1em}\widehat{{\varvec{y}}^{3}}={\varvec{f}}_{{\varvec{\uptheta\:}}_{3}}\left({\varvec{x}}_{\varvec{a}\varvec{d}\varvec{v})}\right)\:,\:\widehat{{\varvec{y}}^{4}}={\varvec{f}}_{{\varvec{\uptheta\:}}_{4}}\left(\stackrel{\sim}{\varvec{x}}\right)$$

The aggregation of the predictions using weighted averaging combination method is modeled as:

$$\:\widehat{{\varvec{y}}_{\text{final}}}={\varvec{w}}_{1}{\varvec{f}}_{{\varvec{\uptheta\:}}_{1}}\left({\varvec{x}}_{\varvec{a}\varvec{d}\varvec{v})}\right)+{\varvec{w}}_{2}{\varvec{f}}_{{\varvec{\uptheta\:}}_{2}}({\varvec{x}}_{\varvec{a}\varvec{d}\varvec{v})}+{\varvec{w}}_{3}{\varvec{f}}_{{\varvec{\uptheta\:}}_{3}}({\varvec{x}}_{\varvec{a}\varvec{d}\varvec{v})})+{\varvec{w}}_{4}{\varvec{f}}_{{\varvec{\uptheta\:}}_{4}}(\stackrel{\sim}{\varvec{x}})$$

$$\:\widehat{{\varvec{y}}_{\text{final}}}={\varvec{w}}_{1}\widehat{{\varvec{y}}^{1}}+{\varvec{w}}_{2}\:\widehat{{\varvec{y}}^{2}}\:+{\varvec{w}}_{3}\widehat{{\varvec{y}}^{3}}+{\varvec{w}}_{4}\widehat{{\varvec{y}}^{4}}$$

the aggregation of the predictions using majority voting method is formulated as:

$$\:\widehat{y\text{final}}=\text{mode}\left(\left({\varvec{f}}_{{\varvec{\uptheta\:}}_{1}}\right({\varvec{x}}_{\varvec{a}\varvec{d}\varvec{v})}),{\varvec{f}}_{{\varvec{\uptheta\:}}_{2}}({\varvec{x}}_{\varvec{a}\varvec{d}\varvec{v})},{\varvec{f}}_{{\varvec{\uptheta\:}}_{3}}\left({\varvec{x}}_{\varvec{a}\varvec{d}\varvec{v})}\right),{\varvec{f}}_{{\varvec{\uptheta\:}}_{4}}\left(\stackrel{\sim}{\varvec{x}}\right)\right)$$

Where g_ϕ_: Denoising autoencoder that reconstructs the input x + x_adv,_$\:\stackrel{\sim}{y}$ are Smoothed labels used during training. Adversarial perturbation is bounded by ϵ,η is the Gaussian noise added for augmentation.

#### Synergy between defense mechanisms

To elucidate the synergy between the defense mechanisms and their complementary strengths within the proposed ensemble defense framework, we outline the roles and contributions of each component:

- **Adversarial training**: This mechanism directly enhances the model’s robustness by training it on adversarial examples, ensuring that the model can effectively handle worst-case perturbations and maintain performance under adversarial conditions.
- **Gaussian augmentation**: By exposing the model to random noise during training, Gaussian augmentation improves the model’s generalization capabilities. It complements adversarial training by addressing small, non-adversarial perturbations that may otherwise degrade performance.
- **Label smoothing**: This technique reduces the model’s overconfidence and improves its calibration by encouraging smoother decision boundaries. As a result, it becomes more challenging for adversarial examples to exploit sharp decision boundaries, thereby enhancing the model’s resilience to adversarial attacks.
- **Denoising autoencoders**: Acting as a preprocessing step, denoising autoencoders removes noise and adversarial perturbations from input data. This enhances the effectiveness of adversarial training and Gaussian augmentation by ensuring that the input data fed into the model is cleansed of adversarial noise, thereby improving overall robustness.

Together, these defense mechanisms form a cohesive ensemble that leverages their complementary strengths to significantly enhance the robustness and performance of the intrusion detection system (IDS) against adversarial threats.

### Platform and tools

We use the Jupiter notebook hosted in Google Collaboratory^21^ to develop our deep learning source code in Python. Google Collaboratory provides an interactive environment for writing and running code in Python and other languages. In addition to being cloud-hosted and equipped with multiple deep learning frameworks and libraries pre-installed to speed up the process of creating machine learning models, Collaboratory also provides powerful GPU features. All the models mentioned earlier were implemented in the TensorFlow and Keres framework. Multiple experiments were performed to evaluate the effectiveness of the proposed defense algorithm. It was examined on the Google Colab platform on an Intel(R) Core (TM) i7- 4600(U) CPU 2.70 GHz personal computer with 8GB RAM. Adversarial examples are produced using the open-source IBM Adversarial Robustness Toolbox (ART) framework^22^.

- Network traffic datasets

To demonstrate the robustness, generalization capability, and scalability of proposed defense mechanisms, the experimental evaluation considered in our paper is carried out on two recent public network traffic datasets, CIC-IDS2017 [36] and CIC-IDS 2018. CIC-IDS2017 is chosen because it is a good representation of large modern network environments. On the other hand, CIC-IDS 2018 is More realistic due to larger scale, encrypted traffic, and modern attacks. Table 1. lists the key differences between the two datasets:

**Table 1:** Key differences between CIC-IDS2017 and CIC-IDS2018 datasets.

Aspect	CIC-IDS2017 [23]	CIC-IDS2018 [31 ]
Size	CIC-IDS2017 (~ 2.8 million records).	CICIDS2018 is significantly larger (~ 16 million records)
Attack Types	Contains 14 attacks and Simulates in a controlled environment.	Includes 7 more modern attacks (e.g.Botnet, infilteration). Simulated in a more dynamic and realistic environment that focuses on encrypted traffic.
Complexity	Simpler traffic patterns, less sophisticated attacks.	It has more complex traffic patterns, making it more challenging and realistic. More sophisticated attacks, reflecting modern threat landscapes
Feature set	80 features (e.g., flow duration, packet size, protocol, etc.)	Similar 80 features, but CICIDS2018 includes refinement and better handling of encrypted traffic.

### Preprocessing the datasets

For training and evaluating our model, we use the CIC-IDS-2017,CIC-IDS-2018 datasets produced by the Canadian Institute of Cyber Security by Lash Kari et al.^23,31^ respectively. CIC-IDS-2017consists of labeled normal and attack traffic data (flow)samples of 15 classes with 79 features, whereas CIC-IDS-2018 consists of labeled normal and attack traffic data (flow)samples of 8 classes with similar 79 features. Various preprocessing steps were performed on the datasets. Preprocessing steps include data cleaning. Encoding categorical features and label encoding, feature standardization (scaling) to limit the values between 0,1, and normalization to remove outliers and enhance performance. To balance the entire dataset before splitting, the data sampling is adopted by selecting 50% only of each class member except those containing less than 2000 instances they are taken entirely.

**Fig. 2:**
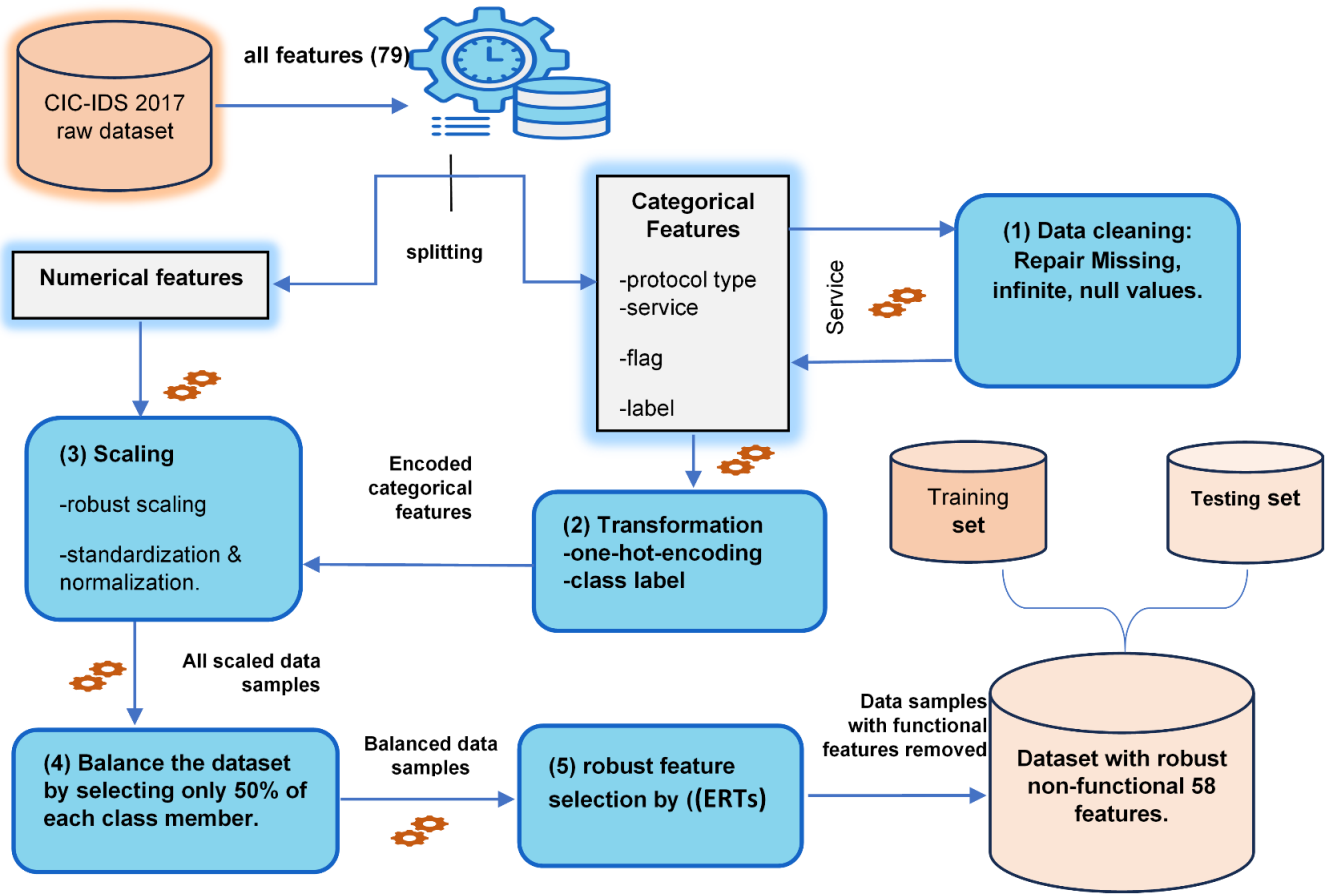
The preprocessing stage data flow diagram (DFD).

**Fig. 3:**
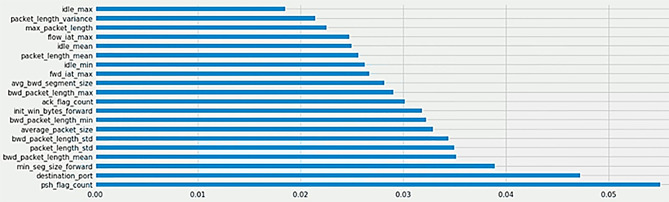
The functional features to be removed from the dataset.

To ensure that the functionality of the network communication channel is preserved while handling the traffic flow samples by the IDS, the non-functional features that do not matter the most are selected for training and testing purposes.in this context, we use the Extremely Randomized Trees (ERTs) classifier to select the most important (functional)features and exclude them. Thus, only data samples with 58 features will be used for training and testing the IDS model. The data flow diagram (DFD) for the entire preprocessing stage is exhibited in Fig. 2. Figure 3. depicts the 20 most prominent features that will be removed.

### Adversarial examples generation

Several experiments have been conducted to generate diverse adversarial samples, encompassing a range of state-of-the-art adversarial attack methodologies, including the Fast Gradient Sign Method (FGSM), Basic Iterative Method (BIM), Deep Fool, Jacobian-based Saliency Map Attack (JSMA), Projected Gradient Descent (PGD), and Carlini & Wagner (C&W) attacks. Table 2 lists the key attack parameters used.

**Table 2:** The key parameters of the generated adversarial attacks.

Attack	Perturbation Strength (ε)	Iteration Count	Norm Constraints
FGSM	0.2	No (single step)	L∞-norm
BIM	0.3	100	L∞-norm
PGD	0.3	100	L∞-norm
C&W	Implicit(minimized by optimization)	9	L_2_-norm
Deep Fool	1e-6	100	L_2_-norm
JSMA	0.1	Not specified	None(feature-based)

For the generation of attack instances, we consider as a substitute model a fully connected feed-forward neural network classifier with an input layer of dimension 58 to accept input samples with only the non-functional features to be perturbed followed by two layers of 100 neurons each. The output layer contains 15 neurons, which correspond to one of 15 possible label classes. We employ a learning rate of 0.01 within 30 training epochs,256 mini-batch size, and categorical cross-entropy loss function. We obtain the same detection ability as the IDS baseline classifier on the clean data samples, with an average F1 score of 0.98.

We implement a feed-forward deep neural network (DNN) based IDs model as a baseline model to evaluate the generated adversarial samples effect before and after applying the adversarial ensemble defense. From the training dataset, we randomly select 40,000 samples (10,000 for each attack) of which 20,000 represent regular traffic and the remaining represent intrusions. From the testing data set we select 20.000 samples (5000 for each attack) The size of perturbation is ranged from 0.1 to 0.3 for keeping the attributes of the original traffic samples, with the mean squared error norm (*p* = 2) serving as the measure metric. The baseline IDS classifier would incorrectly identify the normal as intrusions if perturbations were added, and vice versa.

### Defense strategies

We begin by evaluating the robustness of the baseline deep learning-based intrusion detection system (IDS) model against the generated adversarial examples without implementing any defense mechanisms. This initial assessment serves as a benchmark to quantify the model’s vulnerability to adversarial attacks in its default state. Subsequently, we examine the impact of integrating multiple defense strategies—label smoothing, Gaussian augmentation and adversarial training —during the training phase to enhance the classifier’s robustness against adversarial samples. Notably, the application of these defense mechanisms, particularly when trained with smoothed labels, results in a significant improvement in the classifier’s accuracy and resilience. This demonstrates the effectiveness of the proposed defense strategies in mitigating adversarial threats and improving the overall performance of the IDS model.

**Fig. 4:**
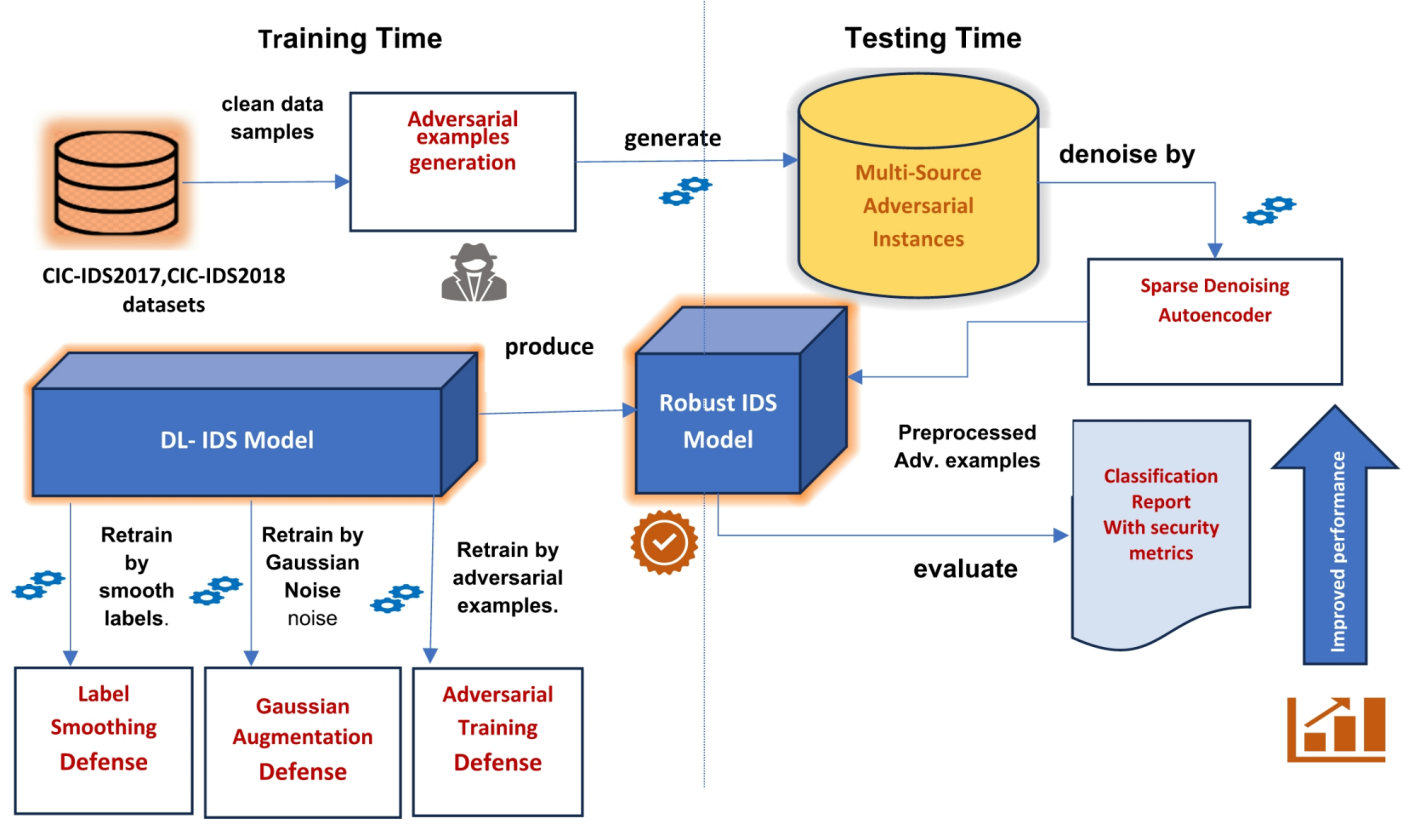
The Proposed Ensemble Defense Method.

As an additional defense strategy implemented during the testing phase, we propose the use of a Denoising Autoencoder (DAE) to serve as a preprocessing framework. This framework is designed to purify or denoise the input traffic flow samples by removing adversarial perturbations before they are fed into the intrusion detection system (IDS) classifier. As outlined in Algorithm 5, the denoising process involves training the DAE to reconstruct clean samples from perturbed inputs. Specifically, the DAE is trained on a combination of clean and adversarial samples, enabling it to learn robust representations that effectively filter out adversarial noise. Once trained, the DAE acts as a denoiser, processing perturbed test samples and producing denoised outputs. The IDS classifier is then evaluated using these denoised samples to assess its robustness against adversarial attacks. Figure 4 illustrates the proposed ensemble defense framework, which integrates this denoising mechanism alongside other defense strategies. The complete ensemble defense algorithm is formalized in Algorithm 1, providing a comprehensive approach to enhancing the robustness of the IDS model.

**Algorithm 1:**
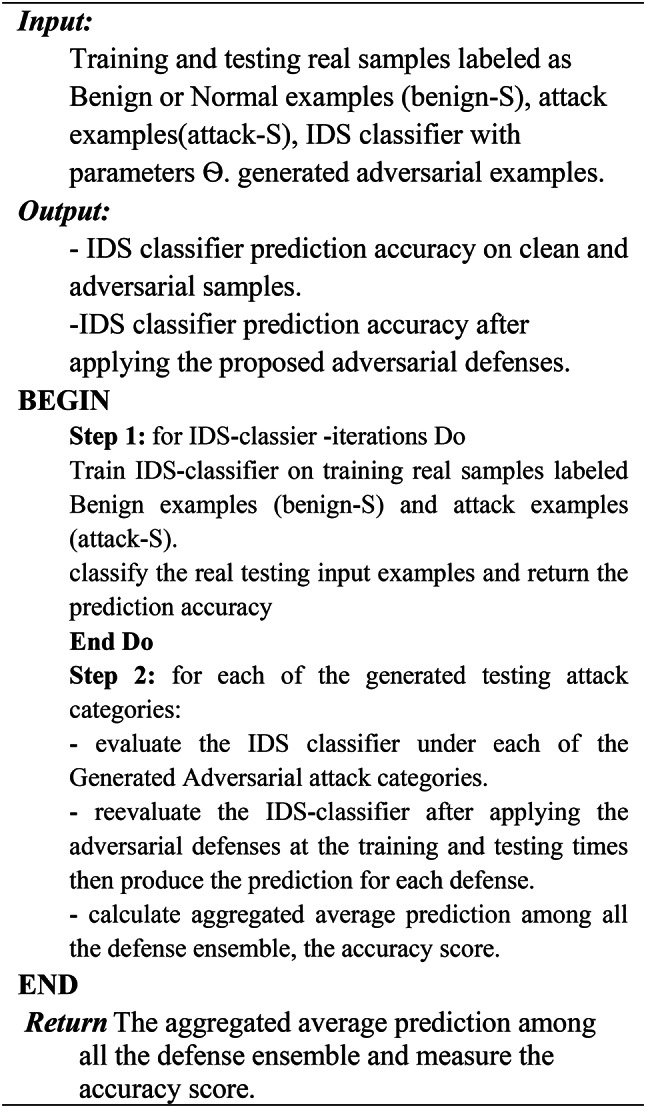
IDS classifier training and evaluation on clean dataset, adversarial examples, and ensemble defense.

As mentioned earlier, the ensemble defense strategy is adopted in two stages. At the training time to enhance the IDS classifier robustness. First, by applying the label smoothing defense by training the IDS classifier with a vector of smooth labels to prevent overfitting, stabilize the training process, and overcome over-confident prediction, as depicted in Algorithm 2. Second, by adopting the Gaussian augmentation defense as described in Algorithm 3, and finally, by using the adversarial training as illustrated in Algorithm 4.

**Algorithm 2:**
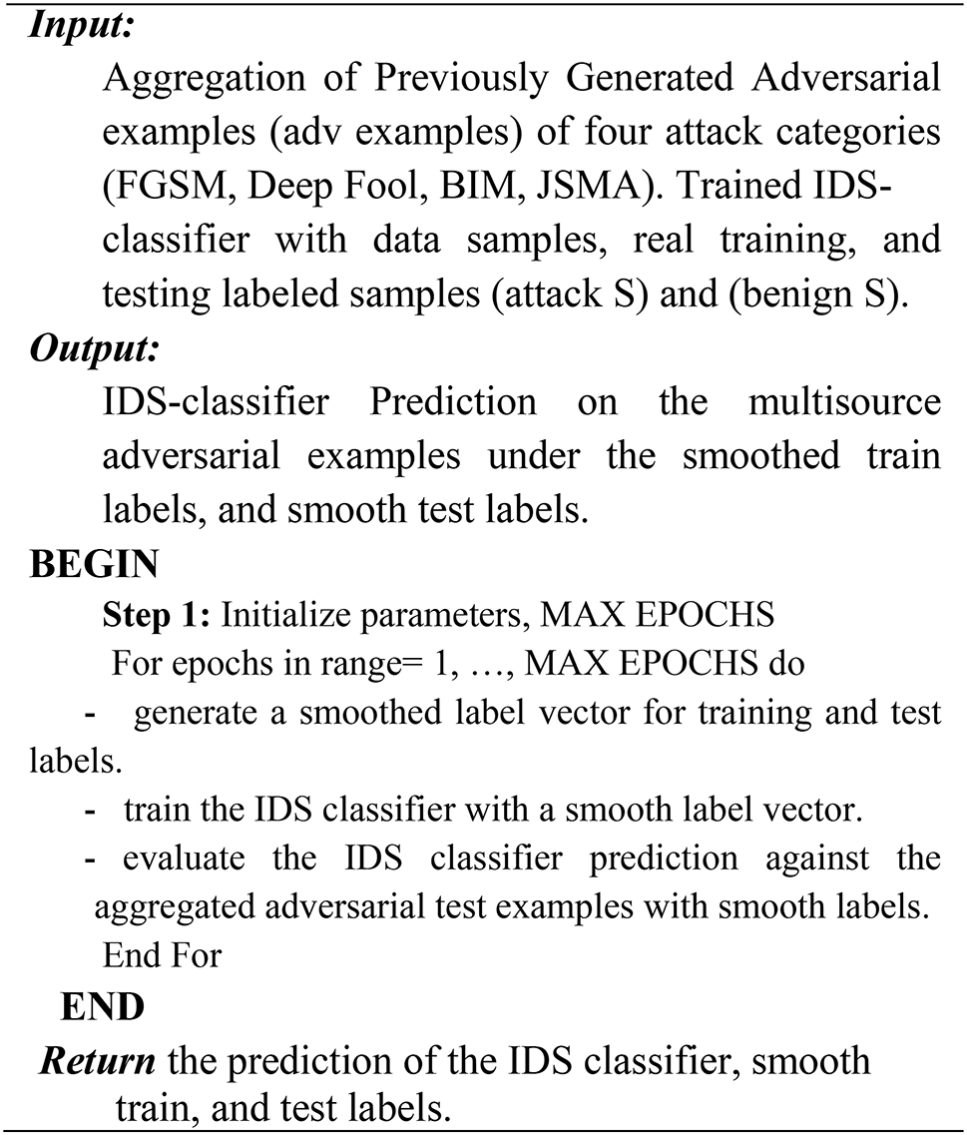
Label Smoothing Defense.

As mentioned in^24^ label smoothing y_ls replaces y_hot with a combination of the uniform distribution and the one-hot encoded label vector y_hot according to Eq. (4).

4 $$\:\mathbf{y}\_\mathbf{l}\mathbf{s}\:=\:(1\:-\:)\:\mathbf{*}\:\mathbf{y}\_\mathbf{h}\mathbf{o}\mathbf{t}\hspace{0.17em}+\hspace{0.17em}\:/\:\mathbf{K}\:$$

**Algorithm 3:**
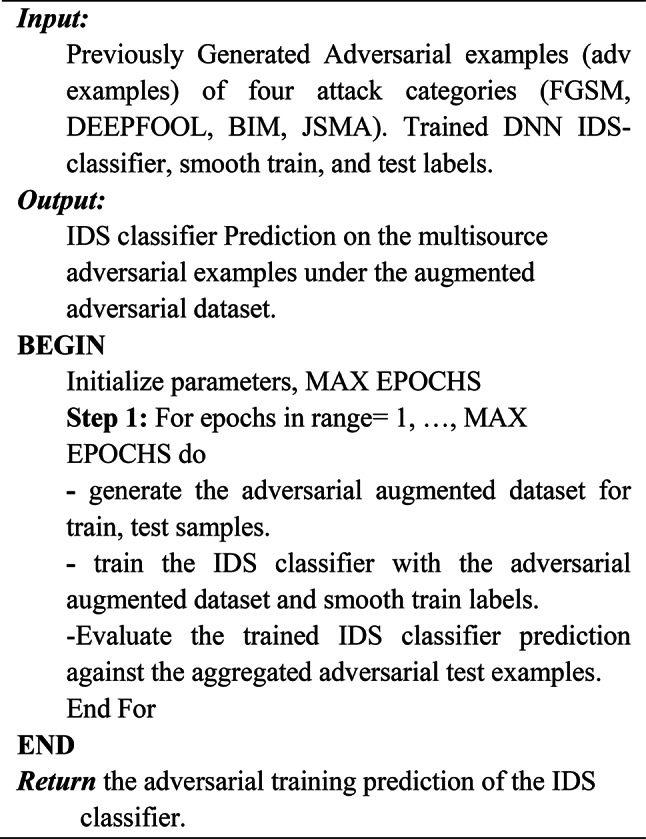
Gaussian Augmentation Defense.

**Algorithm 4:**
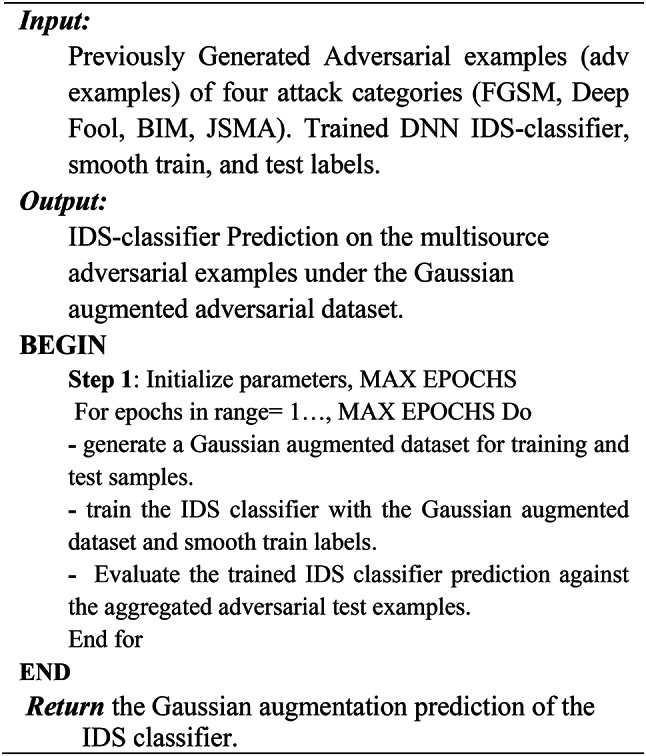
Adversarial Training Defense.

where α is a hyperparameter that controls the degree of smoothing and K is the number of label classes. The original one-hot encoded y_hot is obtained if α = 0, whereas we obtain the uniform distribution if α = 1.

Gaussian augmentation (GA) defense promotes the IDS classifier robustness against adversarial instances. It involves training the IDS classifier with dataset of the original data samples as well as newly created noisy samples (augmentation = True) with the same size as the train and test samples. As illustrated in Eq. (5)^15^

5 $$\:\mathbf{M}\mathbf{i}\mathbf{n}\:\:\mathbf{E}\:(\mathbf{x},\:\mathbf{y})\:\sim\:\:\mathbf{D}\:\mathbf{E}\:\mathbf{x}\:\sim\:\:\mathbf{N}\:(0,\:2)\:\mathbf{J}\:(,\:\mathbf{x}\hspace{0.17em}+\hspace{0.17em}\mathbf{x},\:\mathbf{y})\:$$

The symbol σ in Eq. (3) denotes the degree of unnoticeable disturbance brought about by GDA. It is a formal depiction of the training objective function for the NIDS model. The expected value over the training dataset is indicated by the expression “E (x, y) ~ D,” while the expectation over perturbations resulting from a normal distribution with a mean of 0 and variance of “σ^2^” is indicated by the term “EΔx ~ N(0,σ^2^).” Reducing the expectation is the goal. It guarantees that the predictions made by the model (‘p(y|x)’) roughly correspond to a Gaussian distribution with a variance of ‘σ^2^′ and a centering around the initial input ‘x’. Through the introduction of variability during training, this technique helps to improve the robustness of the model and its ability to handle perturbed input data.

Adversarial Training defense expands the training and testing dataset with the adversarial examples and then trains the IDS classifier with the generated samples.

## Experimental results and evaluation

In the subsections that follow the evaluation metrics that assess the IDS classifier performance on CIC-IDS2017 under different settings are explained. Next, the DNN-based IDS architecture characteristics and the hyperparameters that control the learning process are clarified. Finally, a Comparison of Adversarial Resilience on deep learning-based IDS performance concerning adversarial free, adversarial threats, adversarial defenses, and ensemble defense is obtained. The experimental results and evaluation of the defense model on CIC-IDS2018 are included in the supplementary file.

### Evaluation metric

To evaluate and interpret the impact of our defense strategies on the IDS classifier performance against the adversarial environment, different metrics are being measured. Among them is the Prediction Accuracy (AC): which refers to the Total number of correctly classified samples from benign and attack samples among all samples and given by Eq. (6):

6 $$\:\varvec{A}\varvec{c}\varvec{c}=\:\frac{\varvec{T}\varvec{P}+\varvec{T}\varvec{N}}{\varvec{T}\varvec{P}+\varvec{T}\varvec{N}+\varvec{F}\varvec{P}+\varvec{F}\varvec{N}}$$

Specificity (or Precision): refers to the number of true positives among samples classified as positive. As formulated in Eq. (7):

7 $$\:\varvec{P}\varvec{e}\varvec{r}\varvec{c}\varvec{i}\varvec{s}\varvec{i}\varvec{o}\varvec{n}=\:\frac{\varvec{T}\varvec{P}}{\varvec{T}\varvec{P}+\varvec{T}\varvec{N}}$$

Sensitivity (or Recall): known as the True Positive Rate (TPR), which is the number of True Positives (TP) among all actual positive samples. Made by Eq. (8):

8 $$\:\varvec{R}\varvec{e}\varvec{c}=\:\frac{\varvec{T}\varvec{P}}{\varvec{T}\varvec{P}+\varvec{F}\varvec{N}}$$

The harmonic mean of precision and recall is the F1-score, which is an effective measurement, formulated in Eq. (9):

9 $$\:\varvec{F}1-\varvec{s}\varvec{c}\varvec{o}\varvec{r}\varvec{e}=\:\frac{2\varvec{*}\varvec{p}\varvec{r}\varvec{e}\varvec{c}\varvec{i}\varvec{s}\varvec{i}\varvec{o}\varvec{n}\varvec{*}\varvec{r}\varvec{e}\varvec{c}}{\varvec{p}\varvec{r}\varvec{e}\varvec{c}\varvec{i}\varvec{s}\varvec{i}\varvec{o}\varvec{n}+\varvec{r}\varvec{e}\varvec{c}}$$

### Architecture characteristics and learning setup

To evaluate the effectiveness of the proposed defense model, a series of experiments were conducted targeting the deep learning-based intrusion detection system (IDS) classifier. A feed-forward deep neural network (DNN) model was trained on a preprocessed clean training dataset. For the CIC-IDS2017 dataset, the training set initially comprised 79 traffic flow features, which were reduced to 58 features using the Extremely Randomized Trees (Extra Trees) classifier as a feature selection technique. This feature reduction step ensures that only the most relevant and discriminative features are retained for model training. The trained DNN-based IDS classifier serves as the baseline model, which is subsequently targeted by adversarial examples to assess its vulnerability. It is worth mentioning that we apply the same framework with the similar settings on the CIC-IDS2018 dataset for generalization capability. The architecture design and hyperparameters of the DNN-based IDS classifier are detailed in Table 3, providing a comprehensive overview of the model configuration used in this study.

**Algorithm 5:**
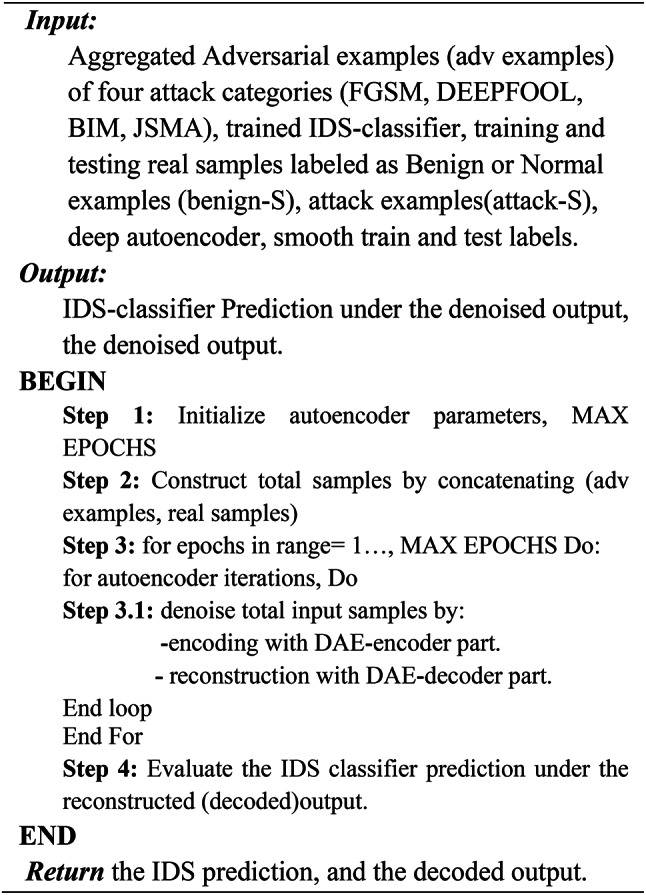
Denoising autoencoder Defense.

**Table 3:** DNN-based IDS classifier architecture design and hyper- parameters.

The number of layers	Three sequential layers, including one input layer with input dimension 58, two hidden layers, and one output layer (512-256-15).
Hidden layers description	The hidden layers are fully connected layers with the Relu activation function. The output layer adopts the SoftMax activation function with Adam optimizer and categorical cross-entropy loss for multi-class traffic classification.
Batch size Learning Rate No. of Epochs Validation Split	128 0.01 30 0.33

### Comparisons and analysis

This section presents a comparative analysis of the adversarial resilience of deep learning-based intrusion detection systems (IDS) when equipped with various adversarial defense mechanisms. The study evaluates the effectiveness of individual and ensemble defense strategies, including adversarial training, Gaussian augmentation, label smoothing, and denoising autoencoders, in mitigating the impact of adversarial attacks. The performance of the IDS model is assessed under both clean and adversarial conditions, with a focus on key metrics such as accuracy, precision, recall, and f1-score, robustness, and resilience to adversarial perturbations. The results highlight the relative strengths and limitations of each defense mechanism, as well as the synergistic benefits of combining them into a unified defense framework using majority voting and weighted average aggregation. This comparison provides valuable insights into the trade-offs and effectiveness of different adversarial defense strategies in enhancing the robustness of deep learning-based IDS models.

- ***Comparison of adversarial resilience in deep learning-based intrusion detection system with respect to adversarial defense mechanisms***

In the following subsections, we analyze and compare the performance of the deep neural network (DNN)-based intrusion detection system (IDS) detector under three distinct scenarios: (1) adversarial-free conditions, (2) adversarial attack conditions, and (3) ablation study to evaluate the effectiveness of individual defense techniques and their combined impact. The ablation study involves training and testing the IDS model with each defense mechanism applied independently. The performance of these individual defense strategies is then compared against the ensemble approach, which integrates all defense mechanisms using majority voting and weighted average aggregation. This comprehensive evaluation aims to quantify the relative contributions of each defense technique and demonstrate the synergistic benefits of combining them into a unified defense framework. We evaluate the proposed defense mechanism over the CIC-IDS2017,CIC-IDS2018.The evaluation results obtained from CIC-IDS 2018 are included in supplementaryS1 through S6.

**Fig. 5:**
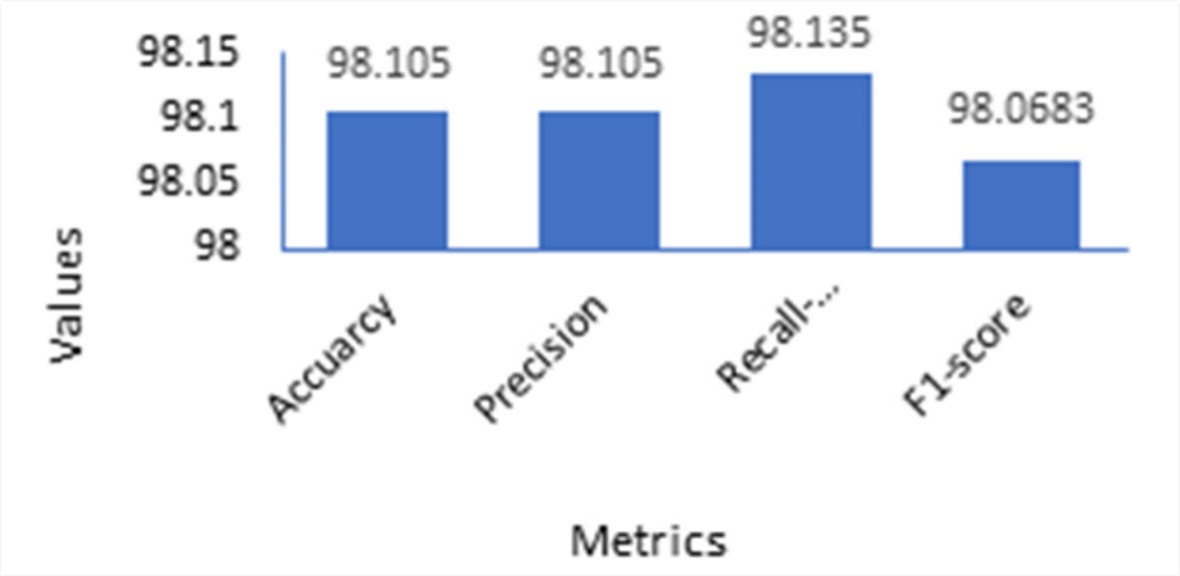
DNN-based IDS Classifier Performance under adversarial free settings.

A. ***DNN-based IDS classifier performance under the adversarial free setting***:

We begin by evaluating the DNN-based IDS detector performance on clean data samples before attacking. Table 4. shows the DNN-based IDS model performance under the adversarial free setting. We observe that the target DNN-based IDS classifier achieves outstanding classification metrics values on clean data samples. Figure 5. Illustrates its performance.

**Table 4:** DNN-based IDS detector performance under adversarial free settings.

Accuracy	Recall	Precision	F1-score
98.11%	98.11%	98.11%	98.068%

- ***B. Adversarial samples’ impact on DNN-based IDS performance***

**Fig. 6:**
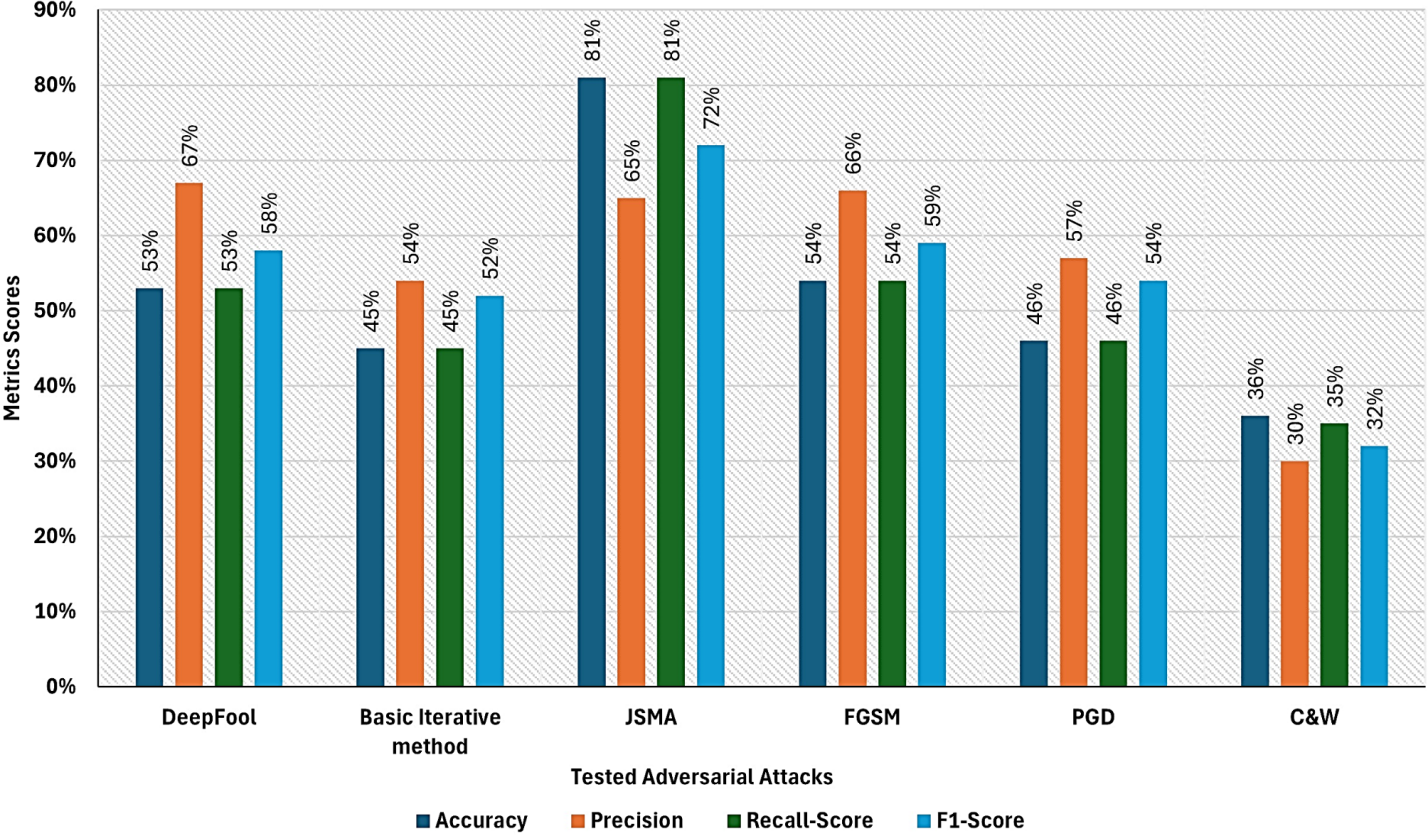
IDS classifier performance Against tested Adversarial Attacks.

Experimental results demonstrate that the efficacy of the target deep neural network (DNN)-based intrusion detection system (IDS) classifier significantly degrades when adversarial samples are introduced into the input data. The initial accuracy of the DNN-based IDS classifier, achieved under adversarial-free conditions, is illustrated in Fig. 5. However, when the model is evaluated using the generated adversarial instances, a substantial decline in prediction accuracy is observed. Specifically, the accuracy drops to 54% under Fast Gradient Sign Method (FGSM) adversarial samples, 53% under DeepFool adversarial samples, 81% under Jacobian-based Saliency Map Attack (JSMA) adversarial samples, 45% under Basic Iterative Method (BIM) adversarial samples, and 36% under Carlini & Wagner (C&W) adversarial samples. These results are summarized in Table 5 and visually represented in Fig. 6, highlighting the vulnerability of the IDS model to various adversarial attack strategies.

- ***C. Ablation study***

As mentioned above; to evaluate the effectiveness of each defense technique and their combined impact, we conduct an ablation study. The study involves training and testing the IDS model with each defense technique individually and comparing their performance against the ensemble approach.

- **Performance of individual techniques**:

- **Label smoothing defense impact on DNN based IDS performance**:

Results listed in Table 6 demonstrate the prediction accuracy of the IDS classifier performance against the tested adversarial attacks improved to almost 82.9When applying the label smoothing defense. This improvement verified that applying label smoothing on training data can reduce overconfidence, prevent overfitting hard labels and hence improve the generalization.

- **Gaussian augmentation defense impact on DNN based IDS performance**:

experiments verified that fitting the ids classifier with gaussian noise augmented training data and the Cross ponding smooth labels can lead to overall improvement to the prediction accuracy against all the tested adversarial attacks and to almost 80.66 when evaluated against the PGD attack as shown in Table 6. Additinally as indicated by Table 7 all the performance measures are improved when applying the gaussian augmentation defense at the training time which enhance the model generalization and make it more robust against noise variations.

- **Adversarial training defense impact on DNN based IDS performance**:

as depicted in Tables 6 and 7 Further improvement in the model performance is achieved by training the IDS model with adversarial examples and the corresponding smooth labels.This is reflected particularly in improvement in accuracy to 80.75% which verify that the model robustness against worst case adversarial perturbations is enhanced and becomes less sensitive to it.

- **Denoising autoencoder defense impact on DNN based IDS performance**:

When evaluating the ids performance on the denoised version of data obtained by using the sparse denoising autoencoder at the testing time the model showed further improvements in robustness against the selected adversarial attacks and security metrics by reconstructing clean inputs from noisy or perturbed inputs. Tables 6 and 7 lists the IDS performance results on the preprocessed input data.

Experiments verified that the ensemble model prediction accuracy against the embedded adversarial examples within the input samples has significantly improved. After using DAE to purify the input samples. The prediction accuracy increased by approximately 83.85% when evaluating the denoised output with the DNN-based IDS model. Tables 6 and 7 lists the IDS performance results on the preprocessed input data.

**Table 5:** DNN-based IDS detector performance against the generated adversarial examples.

Attack	Measure			
	Accuracy (%)	Precision (%)	Recall (%)	F1-score (%)
DF	53	67	53	58
Bim	45	54	45	52
PGD	46	57	46	54
C&W	36	30	35	32
JSMA	81	65	81	72
FGSM	54.5	66	54	59

**Fig. 7:**
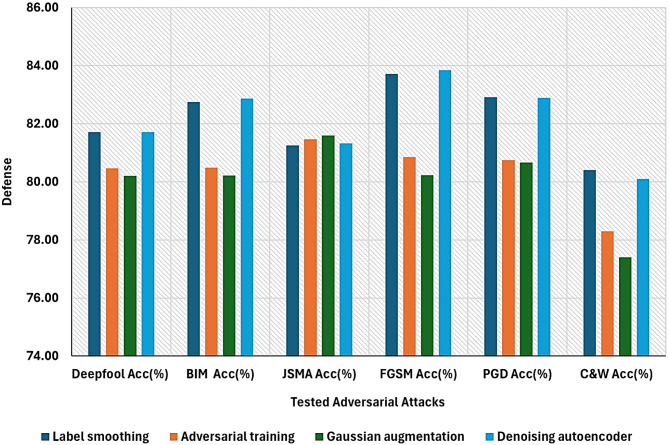
post adversarial defenses Evaluation on IDS classifier performance against the tested adversarial attacks.

- ***Performance of the ensemble defense model***:

Table 7 shows the DNN-based ID classifier accuracy after adopting the proposed defenses. We begin by analyzing the IDS classifier performance when applying the label smoothing defense followed by applying Gaussian augmentation and adversarial training defenses at the training time to improve the adversarial robustness of the IDS classifier. Additionally, at the testing time the perturbed input is denoised by the sparse denoising autoencoder, and the IDS classifier performance is measured against the denoised samples. Figure 7 clarifies the results more. Besides, to take full advantages of using an ensemble defense rather than relying on individual defense mechanisms to combat adversarial attacks the defense models predictions are aggregated using the majority voting and the weighted average combination methods and evaluated by the DNN based IDS classifier.to achieve the optimal performance of the IDS classifier when applying ensemble defense to mitigate against the adversarial threats the majority voting prediction is optimized with soft voting, and the weighted average prediction is optimized with **Bayesian optimization**. Table 8 summarizes the improvement in accuracy score obtained by the ensemble defense aggregation method.

**Table 6:** *DNN-based* IDS detector defense ability after applying the ensemble defense mechanisms.

Defense	Adversarial Attacks					
	Deepfool Acc(%)	BIM Acc(%)	JSMA Acc(%)	FGSM Acc(%)	PGD Acc(%)	C&W Acc(%)
Label smoothing	81.71	82.75	81.25	83.71	82.91	80.4
Adversarial training	80.47	80.49	81.47	80.85	80.75	78.3
Gaussian augmentation	80.21	80.22	81.59	80.23	80.66	77.4
Denoising autoencoder	81.71	82.87	81.33	83.85	82.89	80.1

**Table 7:** IDS performance measures obtained by evaluating the defense mechanisms against the multisource adversarial samples.

Defense mechanism	Measure			
	Accuracy (%)	Precision (%)	Recall (%)	F1- score (%)
Label smoothing	85.9	81.41	87.23	84.2
Gaussian augmentation	79.8	80	79.68	79.8
Adversarial training	80.25	80.75	79.95	78.7
Denoising autoencoder	84.8	82,2	81.87	82.87
Ensemble defense model	84.35	82.56	84.57	83.55

**Table 8:** Accuracy score of majority voting and weighted average aggregation methods and their optimized versions.

Ensemble defense	Accuracy score
Majority voting Ensemble defense	84.35
Optimized majority voting ensemble	87.49
Weighted average ensemble defense	84.45
Optimized weighted average ensemble	86.11

- **Optimizing ensemble defense model aggregation**:

implementing **soft voting** alongside **majority voting** in an ensemble defense model for an intrusion detection system (IDS) can significantly improve the ids performance in fighting the adversarial samples by leveraging both the confidence scores obtained by (soft voting) and the consensus of predictions obtained by (majority voting). Table 8. depicts the improvements achieved in the IDS accuracy score after applying the soft voting technique alongside the majority voting combination method on the ensemble defense model.

As another optimization direction, we use **Bayesian optimization** for optimizing the performance of the weighted average aggregation of an ensemble defense.model,.Bayesian optimization efficiently searches for optimal weights to maximize the accuracy of ensemble defense model.we run the optimization function with 50 iterations and selecting 100 initial random points. At each iteration we set up the optimizer with the scoring function and the parameter bounds that define the limits on the weights. The process continues until we find the optimal weights that maximize the performance of the weighted average ensemble defense model. Table 8. lists the optimized weighted average aggregation accuracy score obtained by IDS classifier against the tested adversarial attacks instances. The supplementary S7. illustrates the accuracy score obtained from both of CIC-IDS2017,CIC-IDS2018 datasets in all tested Scenarios.

- **The Matthews Correlation Coefficient (MCC)** is a robust metric for evaluating the performance of binary classification models, especially in cases of imbalanced datasets. It ranges from **− 1 to + 1**, where **+ 1** indicates perfect prediction,**0** indicates random prediction or no better than random, and**-1** indicates total disagreement between prediction and observation(total inverse prediction).

For adversarial robustness, MCC is particularly useful because it accounts for all four values in the confusion matrix (TP, FP, FN, TN) and provides a balanced measure even when the classes are imbalanced. If the **IDS (Intrusion Detection System) model is a multiclass classification model**, the **Matthews Correlation Coefficient (MCC)** can still be used, but it needs to be generalized to handle multiple classes. The **multiclass MCC** is an extension of the binary MCC and provides a single metric to evaluate the performance of a multiclass classifier. It accounts for all classes and is particularly useful for imbalanced datasets.

Since our primary goal is to evaluate the adversarial robustness of DNN based IDS model after adopting the ensemble defense methods we reduce the classification task into binary classification for simplicity. The formula for MCC is:

$$\:MCC=TP\cdot\:TN-FP\cdot\:FN/\sqrt{\left(TP+FP\right)\left(TP+FN\right)\left(TN+FP\right)\left(TN+FN\right)}$$

**Table 9:** Matthew’s correlation coefficient (MCC) for each defense method.

Defense Method	MCC
Label Smoothing Defense	0.698
Gaussian Augmentation Defense	0.596
Adversarial Training Defense	0.608
DAE Defense	0.680

Table 9 lists Matthews Correlation Coefficient (MCC) for each defense method, Label Smoothing Defense has the highest MCC (0.698), indicating the best adversarial robustness among the evaluated methods. Gaussian Augmentation Defense has the lowest MCC (0.596), indicating relatively low robustness. Adversarial Training Defense and DAE Defense show moderate robustness with MCC values of 0.608 and 0.640, respectively.

## Discussion and comparison with related works

The proposed defense strategy fits into the category of adversarial detection by building up a robust IDS detector. The main aim of the study is to boost the IDS detector’s adversarial robustness through two major phases. The first phase encompasses multi-source adversarial retraining, gaussian noise augmentation, and label smoothing for an improved training process. The second phase promotes the ID’s robust detection further by denoising the adversarial input samples before being fed into it. In contrast with other defense techniques in^13,14^, and^26^ that use the same CIC-IDS 2017dataset, massive adversarial retraining enhances the IDS detector robustness represented by robust detection accuracy but may degrade initial detector standard detection performance (standard accuracy) on clean data and unseen patterns of input samples. Moreover, selecting only the robust nonfunctional feature for adversarial generation also supports this interpretation and explains why the techniques mentioned above may produce higher detection performance.

Other criteria that may affect the detection performance in comparison with the above-mentioned techniques are the adversarial attack strength, type, settings especially the amount of perturbation, the dimension of the input samples to be perturbed, and the testing data size.

Despite the later differences mentioned with other techniques we still obtain a reasonably close detection accuracy that reaches 84.34%.,84.45% respectively by when majority voting and weighted average aggregation fusion rules are applied to our ensemble defense. The reason behind that may be because of denoising the tested input samples before being fed into the IDS detector which is considered an extra layer of defense at testing time.

Using an **ensemble defense** rather than relying on individual defense mechanisms to combat adversarial attacks in Intrusion Detection Systems (IDS) offers several significant advantages. These advantages stem from the diversity, robustness, and complementary strengths of multiple defense strategies working together.key benefits of this approach are (1) enhanced robustness against adversarial attacks due to:

- **Diverse defense mechanisms**: An ensemble combines multiple defense strategies (e.g., adversarial training, Gaussian augmentation, label smoothing, and denoising autoencoders), each targeting different vulnerabilities in the model. This diversity makes it harder for adversaries to craft perturbations that can bypass all defenses simultaneously.
- **Reduced single point of failure**: Individual defenses may have specific weaknesses that adversaries can exploit. An ensemble mitigates this risk by ensuring that even if one defense fails, others can still detect or neutralize the attack.

and (2)improved detection accuracy due to Complementary Strengths because different defense mechanisms excel at detecting and mitigating the effect of different types of adversarial perturbations. For example: adversarial training improves robustness against known attack patterns, denoising autoencoders can filter out noise and perturbations in input data, and label smoothing reduces overconfidence in predictions, making the model less sensitive to hard labels.

To the best of our knowledge, this work introduces the first adversarial ensemble defense strategy designed for multi-class classification in Intrusion Detection System (IDS) detectors, utilizing the CIC-IDS2017 and CIC-IDS2018 benchmark datasets. Unlike conventional approaches that simplify the task to binary classification, our method preserves the granularity of attack types, offering a more intuitive and practical solution for real-world intrusion detection scenarios. The effectiveness of our model is demonstrated in Table 10, which provides a comparative analysis against state-of-the-art models on the CIC-IDS2017 dataset, highlighting the advancements achieved by our approach.

**Table 10:** Comparing different models with our model.

Model	Comparison criteria		
	Attack type	Defense	Measurements(M) and advancement(A)
Our model	FGSM, Deepfool, BIM, JSMA	Ensemble defense approach	The average aggregated prediction accuracy of the defense ensemble improved to 84.34%. 26.09%(Accuracy)(A)
^25^ TAD (Debicha et al., 2023)	FGSM, PGD, Deepfool	Transfer learning-based adversarial detector	74.50% AT (Accuracy)(M) 83.67% TAD (Accuracy) (M)
^26^ Deep Neural Network (Roshan et al., 2023)	FGSM, JSMA, CW, PGD	Adversarial training ((AT)	15.90% (Accuracy) (A) 22.32% (F1-Score) (A)
^14^ RAIDS (Sarıkaya et al., 2023)	GAN-PGD	Reconstruction error	99%Accuracy (M), 1.00%f1-Score (M)

## Conclusion and future work

In this work, we investigate the impact of an adversarial ensemble defense on enhancing the robustness of deep neural network (DNN)-based intrusion detection systems (IDS) against multi-source adversarial attacks. The proposed ensemble defense integrates multiple adversarial mitigation strategies, including label smoothing, Gaussian augmentation, and adversarial training with smooth labels during the training phase. During the testing phase, a deep sparse autoencoder is employed to denoise adversarial samples, serving as an additional layer of defense to purify input samples from adversarial perturbations before they are fed into the DNN-based IDS classifier for evaluation. This preprocessing step ensures that the input data is cleansed of adversarial noise, thereby improving the system’s resilience. The proposed ensemble defense not only significantly enhances the robustness and prediction accuracy of the IDS in resisting adversarial attacks but also maintains high accuracy on clean data samples. Furthermore, it leverages the strengths of ensemble learning, which combines the diversity, robustness, and complementary capabilities of multiple defense mechanisms to achieve superior performance compared to individual defense strategies. A key feature of the proposed ensemble defense is its ability to preserve the functionality of traffic features through robust feature selection, ensuring that the integrity of the data is maintained throughout the detection process. Future research directions include testing the model under various adversarial attack settings, evaluating it with different machine learning (ML) and DNN-based IDS architectures, and validating its performance on other benchmark IDS datasets. Additionally, we aim to explore emerging adversarial attack strategies, investigate new mitigation techniques to further enhance the resilience of ML-based IDS models, and develop advanced methods for robust feature selection.

## Electronic supplementary material

Below is the link to the electronic supplementary material.

Supplementary Material 1

## Data Availability

The Data used within this manuscript is obtained CICIDS 2017 Intrusion Detection Evaluation Dataset by the Canadian Institute for Cybersecurity available at https://www.unb.ca/cic/datasets/ids-2017.html, and the CICIDS2018 Dataset available at: https://www.unb.ca/cic/datasets/ids-2018.html.

## Abbreviations

Acc: Accuracy
BIM: Basic Iterative Method
CNN: Convolutional Neural Network
DT: Decision Tree
DA: Deep Autoencoder
DL: Deep Learning
DNN: Deep Neural Network
DF: Deepfool
DDoS: Distributed Denial of service
ERTs: Extremely Randomized Trees
FN: False Negative
FP: False Positive
FPR: False Positive Rate
FGSM: Fast Gradient Sign Method
GDA: Gaussian Data Augmentation
GAN: Generative Adversarial Networks
GD: Gradient Descent
HTTP: Hyper Text Transfer Protocol
ICMP: Internet Control Message Protocol
CIC-IDS: Intrusion Detection Evaluation Dataset by the Canadian Institute for Cybersecurity
IDS: Intrusion detection systems
JSMA: Jacobian-based Saliency Map Attack
LightGBM: Light Gradient-Boosting Machine
LR: Logistic Regression
LSTM: Long-Short Term Memory
ML: Machine Learning
MSE: Mean Squared Error
MLP: Multi-Layer Perceptron |
RFs: Random Forests
Rec: Recall
SDN: Software Defined Networks
SS: Standard Scaler
SVM: Support Vector Machine
TCP: Transmission Control Protocol
TN: True Negative
TP: True Positive
TPR: True Positive Rate
UDP: User Datagram Protocol
