# Supplementary material for: An enhanced ensemble defense framework for boosting adversarial robustness of intrusion detection systems
Source: Sci Rep. 2025 Apr 23;15:14177. doi: 10.1038/s41598-025-94023-z (PMC12019570; doi:10.1038/s41598-025-94023-z)
Supplement: Supplementary file 1 — Supplementary Material 1 [file 41598_2025_94023_MOESM1_ESM.docx]

Table S1:DNN -based IDS classifier performance on clean data samples using CIC-IDS -2018 dataset.

**Table S1. DNN-based IDS detector performance under adversarial free settings.**

| Accuracy | Recall | Precision | F1-score |
| --- | --- | --- | --- |
| 99.%98 | 99.98% | 99.98% | 99.99% |

Table S2:DNN -based IDS classifier performance on adversarial attack samples using CIC-IDS -2018 dataset.

**Table S2. DNN-based IDS detector performance against the generated adversarial examples.**

| Attack | Measure | | | |
| --- | --- | --- | --- | --- |
|  | **Accuracy (%)** | **Precision**  **(%)** | **Recall**  **(%)** | **F1- score**  **(%)** |
| DF | 34.88 | 12.17 | 34.88 | 18.04 |
| Bim | 36.28 | 13.164 | 36.28 | 19.31 |
| Jsma | 36.38 | 13.23 | 36.38 | 19.41 |
| PGD | 36.21 | 12.98 | 36.11 | 19.11 |
| Fgsm | 37.4 | 13.98 | 37.4 | 20.36 |
| C&W | 37.15 | 13.80 | 37.15 | 20.125 |


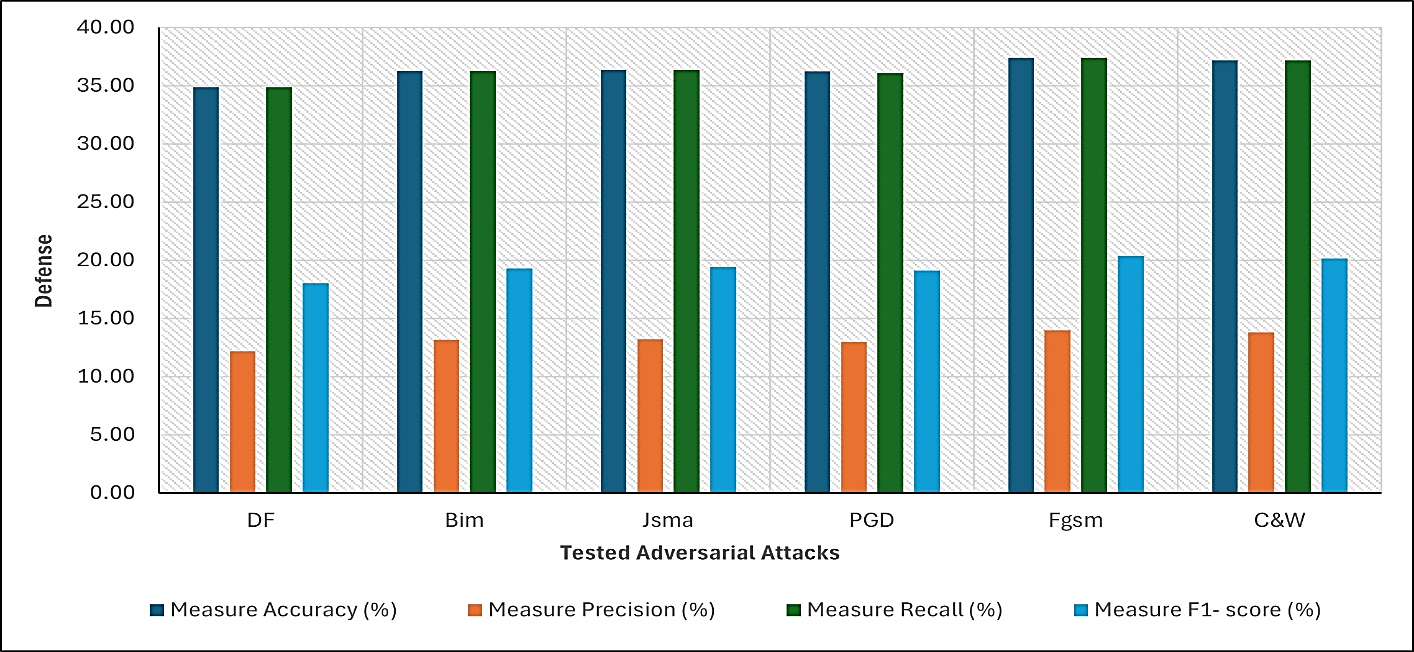


**Figure S1. IDS classifier performance metrics against the tested adversarial attacks .**

Table S3:DNN -based IDS classifier performance after applying the adversarial defenses using CIC-IDS -2018 dataset.

**Table S3. DNN-based IDS detector prediction accuracy after applying adversarial defense.**

| Defense | Adversarial Attacks | | | | | |
| --- | --- | --- | --- | --- | --- | --- |
|  | **Deepfool**  **Acc(%)** | **BIM**  **Acc(%)** | **JSMA**  **Acc(%)** | **FGSM**  **Acc(%)** | **PGD**  **Acc(%)** | **CW**  **Acc(%)** |
| Label smoothing | 94.51 | 96.55 | 93.25 | 96.71 | 96.21 | 91.40 |
| Adversarial training | 92.47 | 91.49 | 91.22 | 90.75 | 91.75 | 89.72 |
| Gaussian augmentation | 91.21 | 91.33 | 90.59 | 93.23 | 91.11 | 89.24 |
| Denoising autoencoder | 94.22 | 96.87 | 93.43 | 96.51 | 95.89 | 90.44 |


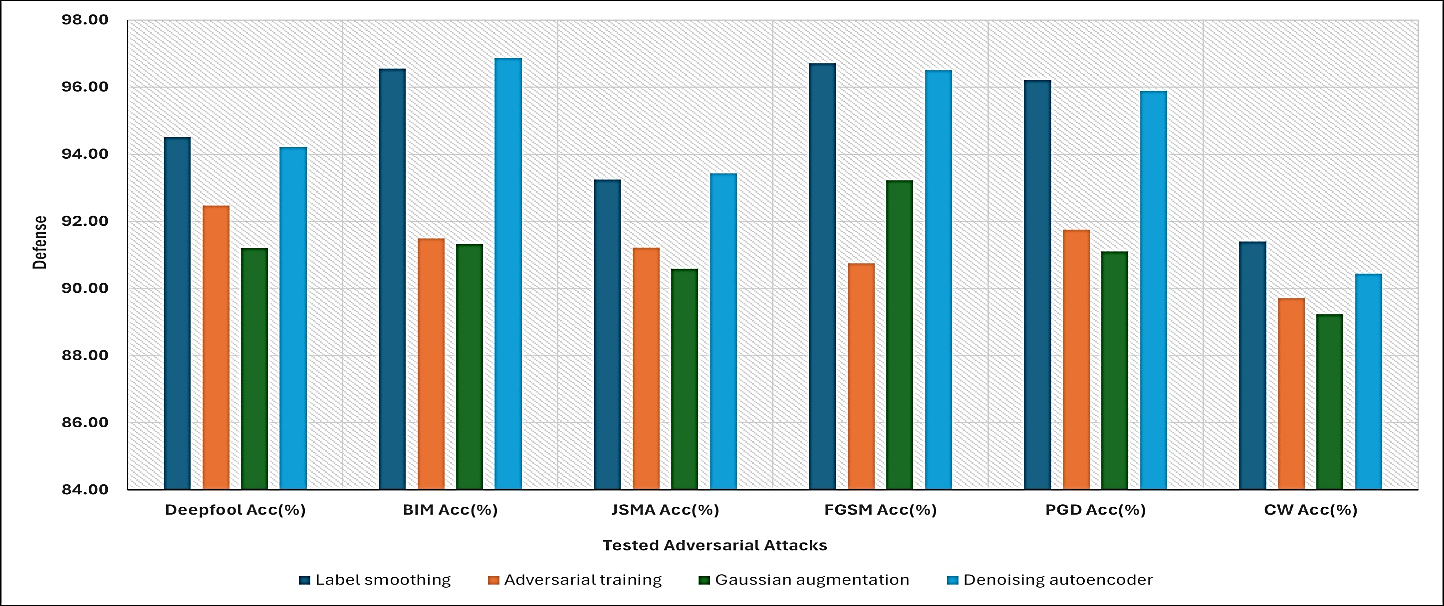


**Figure S2. post adversarial defenses evaluation of IDS classifier accuracy score against the tested adversarial attacks.**

Table S4:The ensemble defense mechanisms performance metrics measures.

**Table S4. The ensemble defense mechanisms performance metrics.**

| Defense mechanism | Measure | | | |
| --- | --- | --- | --- | --- |
|  | **Accuracy (%)** | **Precision**  **(%)** | **Recall**  **(%)** | **F1- score**  **(%)** |
| Label smoothing | 95.9 | 93.41 | 95.87 | 84.2 |
| Gaussian augmentation | 91.65 | 90.34 | 91.58 | 79.8 |
| Adversarial training | 92.25 | 91.75 | 92.24 | 78.7 |
| Denoising autoencoder | 95.8 | 93,81 | 96.77 | 82.87 |
| Ensemble defense model | 96.35 | 94.56 | 96.34 | 83.55 |

Table S5:Accuracy score of the majority voting / weighted average ensemble defense versus optimized majority voting/weighted average ensemble defense.

**Table S5. accuracy score of ensemble defense obtained by majority voting / weighted average aggregation methods.**

| Ensemble | Accuracy score |
| --- | --- |
| Majority voting Ensemble defense | 96.35 |
| Optimized majority voting ensemble | 98.78 |
| Weighted average ensemble defense | 96.47 |
| Optimized weighted average ensemble | 98.74 |

Table S6:Matthews Correlation Coefficient (MCC) for Each Defense Method on CIC-IDS2018 dataset.

**Table S6. Matthews Correlation Coefficient (MCC) for Each Defense Method on CIC-IDS2018 dataset.**

| **Defense Method** | **MCC** |
| --- | --- |
| **Label Smoothing Defense** | 0.762 |
| **Gaussian Augmentation Defense** | 0.638 |
| **Adversarial Training Defense** | 0.694 |
| **DAE Defense** | 0.741 |

Table S7: Accuracy score obtained from both of CIC-IDS2017,CIC-IDS2018 datasets.

**Table S7. accuracy score obtained from both CIC-IDS2017,CIC-IDS2018 datasets.**

| **Scenario** | **CIC-IDS2017 dataset**  **Accuracy Score(%)** | **CIC-IDS2018 dataset**  **Accuracy Score(%)** |
| --- | --- | --- |
| **Adversarial Free** | 98.11 | 99.98 |
| **Adversarial Threat** | 52.58 | 36.38 |
| **Ensemble Defense**  **MV(majority voting)**  **WA(weighted average)** | 84.35(MV),84.45(WA) | 96.35 (MV),96.74(WA) |
| **Optimized Ensemble Defense**  **MV(majority voting)**  **WA(weighted average)** | 87.86 (MV),86.11(w a) | 98.78(MV),98.74(WA) |
